# Progression to type 2 diabetes mellitus and associated risk factors after hyperglycemia first detected in pregnancy: A cross-sectional study in Cape Town, South Africa

**DOI:** 10.1371/journal.pmed.1002865

**Published:** 2019-09-09

**Authors:** Tawanda Chivese, Shane A. Norris, Naomi S. Levitt

**Affiliations:** 1 Chronic Disease Initiative for Africa, Department of Medicine, Faculty of Medicine and Health Sciences, University of Cape Town, Cape Town, South Africa; 2 SAMRC/Wits Developmental Pathways for Health Research Unit, Department of Paediatrics, Faculty of Medicine and Health Sciences, University of the Witwatersrand, Johannesburg, South Africa; Peking University First Hospital, CHINA

## Abstract

**Background:**

Global data indicate that women with a history of hyperglycemia first detected in pregnancy (HFDP) are at up to 7 times risk of progressing to type 2 diabetes mellitus (T2DM) compared with their counterparts who have pregnancies that are not complicated by hyperglycemia. However, there are no data from the sub-Saharan African region, which has the highest projected rise in diabetes prevalence globally. The aim of this study was to determine the proportion of women who progress to T2DM and associated risk factors 5 to 6 years after HFDP in Cape Town, South Africa.

**Methods and findings:**

All women with HFDP, at a major referral hospital in Cape Town, were followed up 5 to 6 years later using a cross-sectional study. Each participant had a 75 g oral glucose tolerance test; anthropometric measurements and a survey were administered. A total of 220 participants were followed up. At this time, their mean age was 37.2 years (SD 6.0). Forty-eight percent (95% CI 41.2–54.4) progressed to T2DM, 5.5% (95% CI 3.1–9.4) had impaired fasting glucose, and 10.5% (95% CI 7.0–15.3) had impaired glucose tolerance. Of the participants who progressed to T2DM, 47% were unaware of their diabetes status. When HFDP was categorized post hoc according to WHO 2013 guidelines, progression in the diabetes in pregnancy (DIP) group was 81% (95% CI 70.2–89.0) and 31.3% (95% CI 24.4–39.3) in the gestational diabetes mellitus (GDM) category. Factors associated with risk of progression to T2DM were; at follow-up: waist circumference (odds ratios [OR] 1.1, 95% CI 1.0–1.1, *p =* 0.007), hip circumference (OR 0.9, 95% CI 0.8–1.0, *p =* 0.001), and BMI (OR 1.1, 95% CI 1.0–1.3, *p =* 0.001), and at baseline: insulin (OR 25.8, 95% CI 3.9–171.4, *p =* 0.001) and oral hypoglycaemic treatment during HFDP (OR 4.1, 95% CI 1.3–12.9, *p =* 0.018), fasting (OR 2.7, 95% CI 1.5–4.8, *p =* 0.001), and oral glucose tolerance test 2-hour glucose concentration at HFDP diagnosis (OR 4.3, 95% CI 2.4–7.7, *p <* 0.001). Our findings have limitations in that we did not include a control group of women without a history of HFDP.

**Conclusions:**

The progression to T2DM in women with previous HFDP found in this study highlights the need for interventions to delay or prevent progression to T2DM after HFDP. In addition, interventions to prevent HFDP may also contribute to reducing the risk of T2DM.

## Abstract 2

Tawanda Chivese reveal a seven times increased rate of developing type 2 diabetes for pregnant women in South Africa if they have hyperglycaemia during pregnancy.

## Author summary

**Why was this study done?:**

- International research shows that when a woman has diabetes detected in pregnancy, which may resolve after the pregnancy, she remains at high risk of future diabetes.
- However, we do not know what proportion of women progress to type 2 diabetes in Africa, because there is no research that has been done before, despite the rapid increase in the number of people with diabetes.

**What did the researchers do and find?:**

- We recalled 220 women 5 to 6 years after they had diabetes first detected in pregnancy and tested them for type 2 diabetes in Cape Town, South Africa.
- We found that almost half of the women (48%) at follow up had type 2 diabetes. Of the women with type 2 diabetes, 47% did not know that they had type 2 diabetes.
- We also found that being obese at follow-up and having higher blood glucose concentrations at the time the women were tested for diabetes in pregnancy (DIP) increased the chances of progressing to type 2 diabetes 5 to 6 years after the pregnancy.

**What do these findings mean?:**

- A large proportion of South African women who have diabetes first detected in pregnancy may develop type 2 diabetes at an early age and within 6 years after the pregnancy.
- It may be necessary to change their lifestyle after the pregnancy so they can reduce the chance of progressing to type 2 diabetes.
- Screening for type 2 diabetes after the pregnancy needs to be more often so women who develop diabetes are diagnosed and receive treatment earlier.
- Further research is needed, because we did not include women with normal blood glucose during pregnancy in this study.

## Background

Sub-Saharan Africa, compared with other regions, is expected to have the greatest increase in the number of people living with diabetes by the year 2040, with more than half the people affected unaware of their diabetes status [1]. Since 2015, diabetes has already risen to be the second leading cause of death, after tuberculosis, in South Africa [2]. The prevalence of obesity, the strongest known risk factor for type 2 diabetes, has increased across the world, and more so in African women, with a recent meta-analysis showing that in this group, mean body mass index (BMI) increased from 22 kg/m^2^ in 1980 to 25 kg/m^2^ in 2014 [3]. In South Africa, the combined obesity and overweight prevalence increased from 29% to 40% in men and 57% to 70% in women during the period of 2002 to 2016 [4,5]. Other drivers of the diabetes epidemic, such as poor nutrition and decreased physical activity, have also increased during the last 2 decades [6]. Further, HIV antiretroviral therapy–induced lipodystrophy may also increase risk of diabetes, especially in women of childbearing age, who are disproportionally affected by HIV, compared with their male counterparts [6]. In view of the current high burden of diabetes and the expected rise in diabetes prevalence, it is imperative to identify populations at elevated risk and introduce risk-lowering interventions.

Women with a history of hyperglycemia first detected in pregnancy (HFDP), including gestational diabetes mellitus (GDM), are at high risk of future development of T2DM [7]. Initially, GDM was defined based on the risk of developing T2DM, but this may have resulted in the inclusion of women with undiagnosed diabetes in the GDM subgroup. Following the recommendations of the International Association of Diabetes and Pregnancy Study Group (IADPSG) [8] and the publication of the findings from the Hyperglycaemia and Adverse Pregnancy Outcomes (HAPO) Study [9]—a multicenter study with participants from 10 countries—WHO [10], in 2013, defined HFDP as either diabetes in pregnancy (DIP) or GDM. According to WHO, GDM is now diagnosed as glucose intolerance in pregnancy with fasting glucose values between 5.1 and 6.9 mmol/L and/or oral glucose tolerance test (OGTT) 2-hour glucose concentrations between 8.5 and 11.0 mmol/L, whereas women with blood glucose values diagnostic of type 2 diabetes first discovered in pregnancy are classified as having DIP. The HAPO Study demonstrated associations between fasting glucose concentrations, as low as 5.1 mmol/L at HFDP diagnosis and adverse fetal outcomes at birth, whereas the HAPO follow-up study [11] and others [12] showed a high risk for T2DM in women in the postpartum period as well as long term and adiposity in their offspring. Notably, neither the HAPO Study nor the follow-up studies included data from an African cohort. Despite the absence of data from African countries, it is expected that lower fasting glucose concentration cut-offs for HFDP diagnosis, in addition to increased awareness and improved screening as well as increasing calls for universal screening for HFDP, may result in a higher prevalence of HFDP worldwide, especially in transitioning populations, such as South Africa. In China, for example, a 4-fold increase in GDM prevalence was noted when universal screening was introduced [13].

Prior to the introduction of the term HFDP, most studies used the term GDM to describe any hyperglycemia first detected during pregnancy. In this article, we use the term HFDP where the studies may have used the term GDM, using older criteria in which the DIP subgroup was possibly included. The prevalence of HFDP varies in different populations, although this is complicated by the use of different diagnostic criteria as well as different screening methods for hyperglycemia during pregnancy [14]. HFDP prevalence from a systematic review of the small number of available studies in Africa ranged from 0% to 14% [15]. Recent studies that used the IADPSG [8] criteria for GDM diagnosis reported prevalence of 8.9% in Nigeria [16], 2.9% in Kenya [17], and in South Africa, 9.1% in Soweto [18] and 25.8% [19] in Johannesburg. The Johannesburg estimate of 25.8% may have included women with DIP and therefore could be an estimate of HFDP. Using the conservative Soweto estimate, if 1 in every 11 pregnancies is complicated by GDM, then public health interventions are required to prevent or delay T2DM in these women post the index pregnancy in South Africa. However, the paucity of data on the prevalence of and associated risk factors for T2DM, in women after GDM, in sub-Saharan Africa, and in South Africa may hinder effective development and planning of interventions and policies.

Data from meta-analyses of studies, mostly from high-income countries, show that women with previous GDM have up to 7-fold risk of developing T2DM [7,20], increased risk of long term cardiovascular disease [7], and for the offspring, increased risk of immediate adverse perinatal as well as future cardiometabolic disease risk [7] compared with those with nondiabetic pregnancies. Further, the risk of progression to T2DM is highest during the period 3 to 6 years post GDM [20]. However, the estimated risks may be overestimates because most of the included studies used older GDM criteria that included women with DIP. In addition, there is a great degree of heterogeneity in the risk for T2DM, with relative risks ranging from 2.7 in Germany to 38.4 in Sweden [14]. The estimates of risk vary by country and within countries, by ethnicity and region, which may be due to differences in the distribution of risk factors of T2DM in different populations. Different follow-up times and different study designs may also contribute to the differences in the risk estimates.

Progression to T2DM post HFDP varies widely, from a low 6% in Australian nonindigenous women [21] to 42% in Indian women [22], using IADPSG criteria. Risk factors for progression also vary widely—ethnicity, increased BMI, family history of T2DM, increased waist circumference and severity of GDM at diagnosis being some of the most frequently identified [23]. In Africa, apart from a single study that followed up 77 women up to 12 weeks post HFDP in Cape Town [24], to our knowledge, there are no data on the progression to T2DM post HFDP or associated risk factors. This study in women 5 to 6 years post HFDP provides the only data to date on the proportion of women who progress to T2DM beyond the postpartum period, as well as factors associated with risk of progression, in Africa, specifically in Cape Town, South Africa. We also investigated the proportion of women who progressed to T2DM in the GDM and DIP groups using the modified WHO 2013 criteria, applied retrospectively.

## Methods

### Study design, setting, and participants

The study was carried according to an ethics approved study protocol (S1 Doc). Data on all women managed for HFDP at Groote Schuur Hospital (GSH) during the period of 1 September 2010 to 31 August 2011 were routinely collected during the index pregnancy [25]. During that time, in the Western Cape province of South Africa, GDM screening and diagnosis was based on the provincial guidelines [26]. Screening was based on selective risk factors—maternal age ≥40 years, BMI ≥40 kg/m^2^, previous GDM, previous fetal birth weight ≥4.5kg, previous unexplained miscarriage, acanthosis nigricans and polycystic ovarian syndrome—whereas GDM was diagnosed using the United Kingdom National Institute for Health and Care Excellence (NICE) 2008 criteria (fasting glucose above 5.5 mmol/L and OGTT 2-hour glucose over 7.8 mmol/L) [27].

A cross-sectional study of the same participants (*n =* 498) was undertaken 5 to 6 years later during the period of 1 January 2016 to 31 Jan 2017. We contacted and invited participants through letters mailed to their last known address, calls to their telephone or cell phone numbers in the hospital record or next of kin and finally, and home visits when all other attempts failed. Women who were pregnant at follow-up were excluded from the study.

### Study procedures and data collected

On the day of testing, participants underwent a standard 75-gram OGTT after fasting for 8 to10 hours. Blood was drawn for glycated haemoglobin A_1c_ (HbA_1C_) as well as glucose and insulin at fasting and 120 minutes post OGTT glucose load. The blood samples were kept on ice, aliquoted within 4 hours of collection, and stored at −80° until analyzed. Participants on treatment for T2DM were not required to do either the OGTT or the HbA_1C_. A trained fieldworker administered a questionnaire (S2 Doc) to obtain sociodemographic information, reproductive history, self-reported personal and family medical history, and psychosocial health and lifestyle factors such as physical activity (modified WHO Global Physical Activity Questionnaire), smoking, and diet using a 2-week food frequency questionnaire.

Height, weight, waist, and hip circumference and blood pressure were measured using standardized procedures. Waist-hip ratio was calculated as the ratio of each participant’s waist circumference to their hip circumference. BMI was grouped according to WHO criteria for underweight (<18.5 kg/m^2^), normal weight (18.5–24.9 kg/m^2^), overweight (25–29.9 kg/m^2^), obese (30–39.9 kg/m^2^), and morbidly obese (>40 kg/m^2^) [28]. Outcomes for each participant were T2DM, impaired fasting glucose (IFG), and impaired glucose tolerance (IGT) using WHO 2006 criteria [29].

### Biochemistry and lab analyses

Plasma glucose was measured using the Randox RX Daytona Chemistry Analyzer. HbA_1C_ was measured using turbidimetric inhibition immunoassay (D10^TM^ Haemoglobin A1c Program; Bio Rad Laboratories, Hercules, CA). The precision and trueness of the Randox RX Daytona Chemistry Analyzer were verified using the Clinical and Laboratory Standards Institute document EP15. Coefficients of variation calculated from running 40 separate samples at 3 different times were 3.0% for glucose and 1.6% for HbA_1C_.

### Sample size and power

The sample size for this study was based on the main aim: to estimate the proportion of participants who progressed to T2DM by the time of follow-up. Most studies found a prevalence of T2DM during the first 5 years after GDM diagnosis between 20% and 50% [12,30]. Using Open Epi sample size calculator for a proportion (http://www.openepi.com/SampleSize/SS), assuming that 35% of our participants would have progressed to T2DM and using the range 20% to 50% from literature (i.e., 15% either side of our assumed proportion), the minimum sample size required was 154. We anticipated difficulties in following-up women in our setting and therefore decided to include all women who we could contact and who agreed to participate.

### Statistical data analysis

All statistical analysis was carried out using Stata 15 statistical software [31]. For all hypothesis testing and comparisons, significance was set at 0.05, whereas 95% CIs were reported for the prevalence of T2DM as well as all odds ratios (ORs). Means and SDs were presented for normally distributed measured variables, medians and interquartile ranges (IQRs) for variables that were not normally distributed, and for categorical variables, frequencies and proportions were reported.

To compare variables between participants who progressed to T2DM and those who did not, chi-squared test and Fischer’s Exact (small frequencies) were used for hypothesis testing for categorical data, whereas the *t* test for independent groups (or Wilcoxon rank sum test if data were not normally distributed) were used to compare measured data.

The analysis for factors associated with T2DM at follow-up was redone after input from journal reviewers, with the main change being using continuous variables (BMI, age, waist and hip circumference) in their raw, and not categorized forms. We carried out a multiple logistic regression model that included variables that have been shown to be associated with risk of T2DM. Variables included from data measured at follow-up were age, anthropometry (BMI, hip and waist circumference), socioeconomic variables (education and employment), comorbidities (self-reported dyslipidemia and high blood pressure), total physical activity from the Global Physical Activity Questionnaire (GPAQ) and family history of diabetes. Variables included from baseline measurements were OGTT glucose concentrations at diagnosis of HFDP and type of treatment for HFDP. Stopping alcohol because of health reasons (*n =* 58 with responses) was not included in the multivariate regression because there were too many missing or “not applicable” data. We also did not include waist-hip ratio in the model because of the very wide 95% CI. Further OGTT 1-hour glucose at HFDP diagnosis was also not included because of its limited clinical utility and because most health facilities in South Africa do not measure it. For logistic regression model diagnostics, we assessed the following: linearity assumption using the Lowes graph, multicollinearity using variance inflation factors, model specification using the C-statistic, and confirmed the fit of the model using the Hosmer-Lemeshow goodness of fit test. We also checked for outliers as well as influential observations.

This study is reported as per the Strengthening the Reporting of Observational Studies in Epidemiology (STROBE) guideline (S1 STROBE Checklist).

### Ethical considerations

The study received ethics clearance from the Human Research Ethics Committees of the University of Cape Town (Reference 656/2015) as well as permission to conduct research at the GSH. Participants gave written informed consent. If found to have undiagnosed T2DM, participants were referred for treatment.

## Results

**Fig 1:**
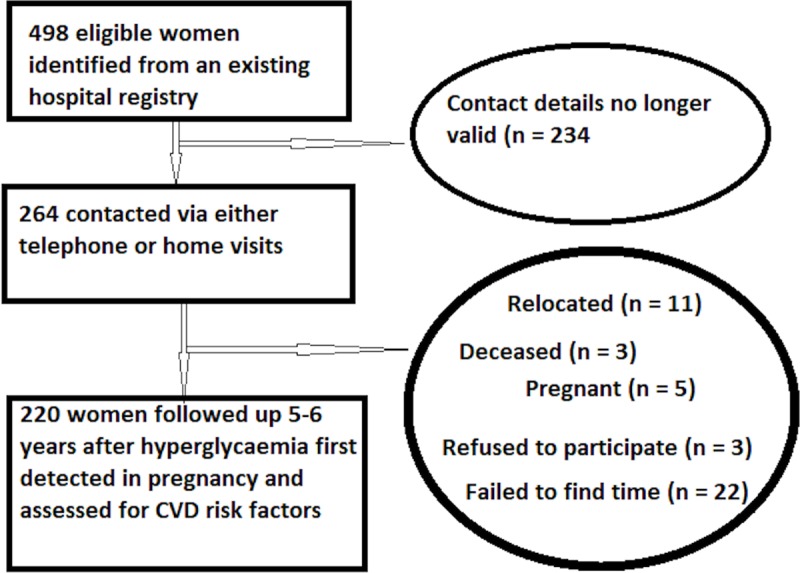
Flow chart of the study. CVD, cardiovascular disease.

Of the 498 eligible women, 220 (44.2%) participated in the follow-up study, 234 (47.0%) could not be contacted, and 44 (8.8%) could not participate (Fig 1). There were no major differences between participants who were followed up and those lost to follow-up, except that participants who followed up had a higher mean BMI at booking and a lower mean OGTT 2-hour glucose concentration at the time of HFDP diagnosis (S1 Table).

### Characteristics of participants

As seen in Table 1, at baseline during the index pregnancy, the mean age was 30.8 years (SD 5.9), most participants (142 [65.4%]) were of mixed ancestry, followed by 68 (31.3%) who self-identified as Black. More than three-quarters had a first-degree family history of diabetes, whereas most participants were either obese (96 [44.9%]) or morbidly obese (49 [22.9%]). Just under a third of the participants (27.7%) received oral hypoglycemic treatment, and 23.6% had insulin therapy for HFDP. At HFDP diagnosis, 70 participants had FPG ≥7 mmol/L and/or 2-hour blood glucose concentrations ≥11.1 mmol/L and were retrospectively classified as DIP, and the remaining 150 (68.2%) had FPG between 5.6 and 6.9 mmol/L and/or 2-hour blood glucose concentrations 7.8 to 11.0 mmol/L, retrospectively, classified as GDM. Our classification of GDM differed from WHO 2013 guidelines in that our cohort did not include women with fasting glucose values lower than 5.5 mmol/L, whereas we included women with OGTT 2-hour blood glucose between 7.8 and 8.4 mmol/L.

**Table 1:** Comparison of baseline characteristics of participants with and without T2DM at follow-up.

Varible	Level	Overall, *N =* 220	Progressed to T2DM, *N =* 105	Did not progress to T2DM, *N =* 115	*P* value
Age, years	Mean (SD)	30.8 (5.9)	31.5 (6.0)	30.3 (5.7)	0.116
Ethnicity, *n* (%)	Black	68 (31.3)	34 (32.7)	34 (30.1)	0.702
	Mixed ancestry	142 (65.4)	67 (64.4)	75 (66.4)	
	Other	7 (3.2)	3 (2.9)	4 (3.5)	
Family history of diabetes, n (%)	Yes	169 (76.8)	81 (77.1)	88 (76.5)	0.913
BMI at booking (*n =* 214), kg/m^2^	Mean (SD)	34.2 (8.2)	35.2 (7.8)	33.3 (8.5)	0.096
BMI at booking categories, *n* (%)	Normal	31 (14.5)	10(9.5)	21(18.3)	0.134
	Overweight	44 (20.0) (17.8)	16 (15.2)	22(19.3)	
	Obese	104 (47.3)	56 (53.3)	46 (40.0)	
	Morbidly obese	48 (21.8)	23 (21.9)	26(22.6)	
HFDP type, *n* (%)	DIP	70 (31.8)	58 (82.9)	12 (17.1)	<0.001
	GDM	150 (68.2)	47 (31.3)	103 (68.7)	
Gestational age at delivery (*n =* 215)	Weeks, median (IQR)	38 (37–39)	38 (37–39)	38 (38–39)	0.001
HFDP treatment, *n* (%)	Oral hypoglycemics	61 (27.7)	38 (36.2)	23 (20.0)	0.007
	Insulin	52 (23.6)	43 (41.0)	9 (7.8)	<0.001
Glucose metabolism at HFDP diagnosis, median mmol/L (IQR mmol/L)	FPG	5.8 (5.1–6.7)	6.4 (5.7–7.2)	5.6 (4.9–5.9)	<0.001
	OGTT 1-hour	10.4 (9.2–11.5)	11.0 (10.0–12.2)	9.8 (8.5–10.6)	<0.001
	OGTT 2-hours	9.0 (8.3–10.4)	10.1 (8.6–11.1)	8.6 (8.1–9.3)	<0.001

*n* is specified for variables with missing data only. All the other variables have complete data.

**Abbreviations:** BMI, body mass index; DIP, diabetes in pregnancy; FPG, fasting plasma gluose; GDM, gestational diabetes mellitus; HFDP, hyperglycemia first detected in pregnancy; IQR, interquartile range; OGTT, oral glucose tolerance test; T2DM, type 2 diabetes mellitus

Table 2 shows the characteristics of the participants at follow-up. At follow-up, the mean age of the participants was 37.2 years (SD 6.0). Most of the participants (167 [75.9%]) had secondary or matric level education. More than two-thirds of the participants were either obese (96 [44.9%]) or morbidly obese (49 [22.9%]).

**Table 2:** Comparison of characteristics at follow-up of participants with and without T2DM.

Variable	Level	Overall, *N =* 220	Progressed to T2DM, *N =* 104	Did not progress to T2DM, *N =* 116	*P* value
Age (years)	Mean (SD)	37.2 (6.0)	37.1 (6.0)	37.3 (5.9)	0.949
Education, *n* (%)	Tertiary	30 (13.6)	9 (8.7)	21 (18.3)	
	Secondary and matric	167 (75.9)	80 (76.2)	87 (75.7)	0.017
	Primary	23 (10.5)	15 (14.6)	6 (5.2)	
Employed, *n* (%)	Yes	108 (49.1)	48 (45.7)	60 (52.2)	0.338
Marital status, *n* (%)	Married	141 (64.1)	68 (64.8))	73 (63.5)	0.843
Hypertension, *n* (%)	Yes	74 (33.6)	42 (40.0)	32 (27.8)	0.056
On treatment for hypertension, *n* (%)	Yes	58 (26.4)	35 (33.3)	23 (20.0)	0.025
Dyslipidemia, *n* (%)	Yes	29 (13.2)	22 (21.0)	7 (6.1)	0.001
On treatment for high cholesterol	Yes	26 (11.8)	20 (19.1)	6 (5.2)	0.002
On ARV treatment, *n* (%)	Yes	8 (3.6)	4 (3.8)	4 (3.5)	0.896
Stopped drinking alcohol due to health reasons, *n* (%) (*n =* 58)	Yes	14 (6.4)	9 (8.6)	5 (4.4)	0.038
GPAQ total physical activity, minutes per week	Median (IQR)	420 (110–1405)	420 (110–1440)	390 (90–1370)	0.890
GPAQ total PA, *n* (%)	≥150 min/week	158 (71.8)	76 (72.4)	82 (71.3)	0.859
**Anthropometry**					
BMI (kg/m^2^)	Mean (SD)	34.9 (8.7)	35.2 (8.9)	34.7 (8.6)	0.705
BMI categories, *n* (%)	Normal	24 (10.9)	14 (13.3)	10 (8.7)	0.329
	Overweight	44 (20.0)	17 (16.2)	27 (23.5)	
	Obese	104 (47.3)	48 (45.7)	56 (48.7)	
	Morbidly obese	48 (21.8)	26 (24.8)	22 (19.1)	
BMI gain (follow-up booking BMI), kg/m2 (*n* = 214)	Median (IQR)	+0.9 (−1.8 to 3.4)	0.0 (−3.0 to 2.8)	+1.6 (1.1–4.0)	0.019
Waist circumference (cm)	Mean (SD)	110.5 (17.6)	111.3 (17.3)	109.7 (18.0)	0.491
Hip circumference (cm)	Mean (SD)	117.3 (16.1)	116.3 (16.5)	118.1 (15.9)	0.416
Waist-hip ratio	Median (IQR)	0.94 (0.89–0.98)	0.95 (0.91–1.01)	0.93 (0.88–0.97)	0.006

**Abbreviations:** ARV, antiretroviral therapy; BMI, body mass index; GPAQ, global physical activity questionnaire; IQR, interquartile range; PA, physical activity; T2DM, type 2 diabetes mellitus.

### Progression to T2DM 5–6 years post HFDP

**Fig 2:**
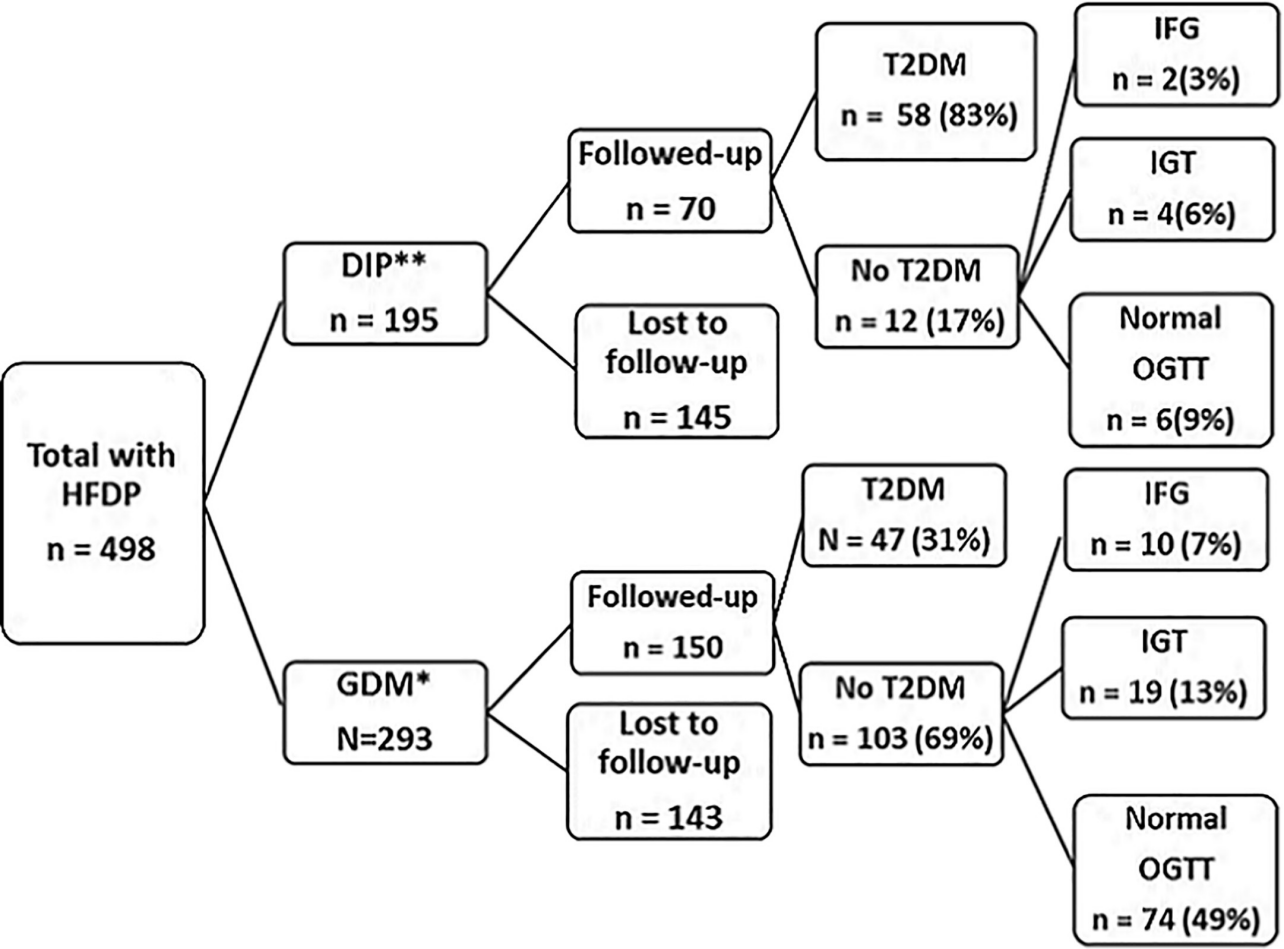
Progression to T2DM by the modified WHO 2013 criteria for GDM. *GDM depicts women who would be categorized as GDM under the WHO 2013 GDM criteria, but this cohort had slightly different cut-offs (fasting glucose between 5.6 and 6.9 mmol/L and/or 2-hour OGTT between 7.8 and 11.0 mmol/L). **DIP depicts women who would be diagnosed as DIP under the WHO 2013 GDM criteria (fasting glucose of at least 7 mmol/L and 2-hour glucose of at least 11.1 mmol/L). DIP, diabetes in pregnancy; GDM, gestational diabetes mellitus; HFDP, hyperglycemia first detected in pregnancy; IFG, impaired fasting glucose; IGT, impaired glucose tolerance; OGTT, oral glucose tolerance test; T2DM, type 2 diabetes mellitus.

At the time of follow-up, 47.7% (*n =* 105, 95% CI 41.2–54.4) progressed to T2DM, of these 47.1% were not previously diagnosed, 12 participants had IFG (5.5%, 95% CI 3.1–9.4), and 23 participants (10.5%, 95% CI 7.0–15.3) had IGT (Fig 2). Using an HbA_1C_ ≥6.5 and an established T2DM diagnosis, the T2DM prevalence was 49.5% (95% CI 42.8–56.2). When HFDP was categorized post hoc, according to the modified WHO 2013 criteria, progression to T2DM in the DIP group was 82.9% (95% CI 72.0–90.1) and 31.3% (95% CI 24.4–39.3) in the GDM category.

### Comparison of participants with and without T2DM at follow-up

Participants who progressed to T2DM compared with those who did not progress had significantly higher median glucose concentrations (in mmol/L) at all times of the OGTT at HFDP diagnosis—fasting: 6.4 (IQR 5.7–7.2 versus 5.6 (IQR 4.9–5.9), *p <* 0.001; 1-hour: 11.0 (IQR 10.0–12.2) versus 9.8 (IQR 8.5–10.6), *p <* 0.001, and 2-hour glucose: 10.1 (8.6–11.1) versus 8.6 (8.1–9.3), *p <* 0.001)—and were more likely to be on either oral hypoglycemic (36.2% versus 20.0%, *p =* 0.007) or insulin therapy (41.0% versus 7.8%, *p <* 0.001) during HFDP (Tables 1 and 2).

At follow-up, compared with participants without T2DM, participants who progressed to T2DM were significantly less likely to have a tertiary level education but more likely to have primary school level education (tertiary: 8.7% versus 18.3%, primary: 14.6% versus 5.2%, respectively, *p* = 0.017), more likely to report having dyslipidaemia (21.0% versus 6.1%, *p =* 0.001), more likely to have stopped drinking alcohol for health reasons (8.6% versus 4.4%, *p =* 0.038), and more likely to have gained less BMI (in kg/m^2^, [median 0.0 (IQR −3.0 to 2.8) versus 1.6 (−1.1 to 4.0), respectively, *p =* 0.019]). Box plots comparing fasting and OGTT 2-hour glucose levels at HFDP diagnosis, waist, and hip circumferences, and waist-hip ratio at follow-up, by T2DM status at follow-up, are shown in S1 Fig, S2 Fig, S3 Fig, S4 Fig, and S5 Fig, respectively.

### Factors associated with progression to T2DM

**Fig 3:**
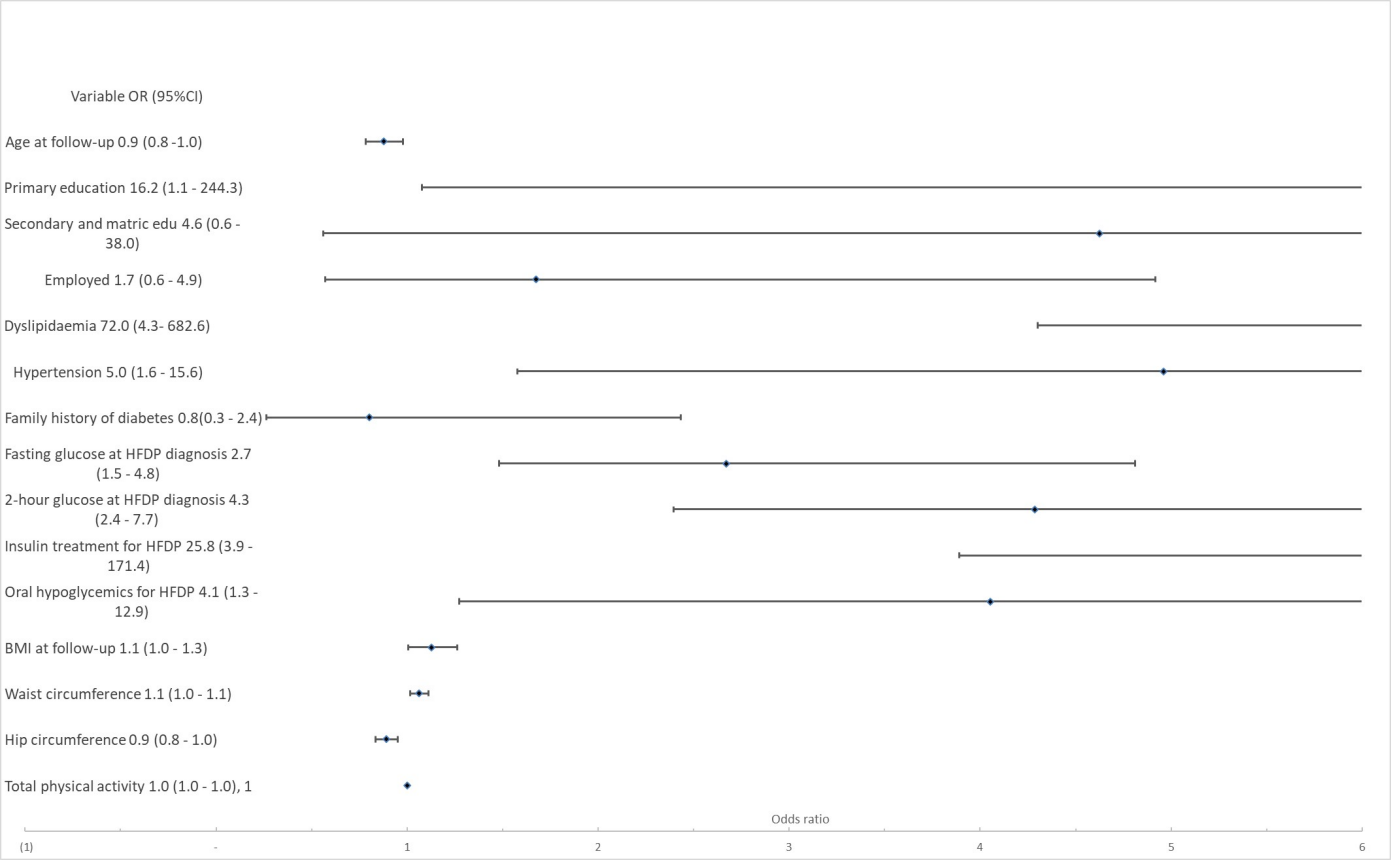
Multiple logistic regression of factors associated with T2DM. Model statistics (observations, 200; LR Chi-squared, 167.4; *p* = 0.000; Pseudo R^2^, 0.401; and log likelihood, 54.6). BMI, body mass index; HFDP, hyperglycemia first detected in pregnancy; matric edu, matric education; LR, likelihood ratio; OR, odds ratio; T2DM, type 2 diabetes mellitus.

Fig 3 shows the results of multiple logistic regression for variables independently associated with T2DM. Baseline variables significantly associated with risk of progression of T2DM were fasting glucose at HFDP diagnosis (OR 2.7, 95% CI 1.5–4.8, *p =* 0.001) and OGTT 2-hour glucose concentration at HFDP diagnosis (OR 4.3, 95% CI 2.4–7.7, *p* < 0.001), oral hypoglycaemic treatment for HFDP (OR 4.1, 95% CI 1.3–12.9, *p =* 0.018), and insulin treatment during HFDP (OR 25.8, 95% CI 3.9–171.4, *p* = 0.001). The following variables measured at the time of follow-up were significantly associated with progression to T2DM: having primary school education only, compared with tertiary education (OR 16.2, 95% CI 1.1–244.3, *p* = 0.044), self-reported dyslipidaemia diagnosis (OR 72.0, 95% CI 7.6–682.6, *p <* 0.001), self-reported hypertension diagnosis (OR 5.0, 95% CI 1.6–15.6, *p =* 0.006), BMI (OR 1.1, 95% CI 1.0–1.3, *p =* 0.001), waist circumference (OR 1.1, 95% CI 1.0–1.1, *p =* 0.007), and hip circumference (OR 0.9, 95% CI 0.8–1.0, *p =* 0.001).

### Logistic regression model diagnostics

The model consisted of 200 observations after the removal of outliers (*n =* 17) and the omission of participants with missing data (*n =* 3). In the final model, the *p*-values for the C-statistic (_hatsq) and the Hosmer-Lemeshow statistic were 0.123 and 0.809, respectively, confirming good fit for the model. There was no significant collinearity because pairwise correlations resulted in variance inflation factors between 1.04 and 1.82. The Lowes graph confirmed the linear model assumption.

## Discussion

Our major findings are that in women 5 to 6 years post HFDP, only 36.4% had normal glucose tolerance; 47.7% had progressed to T2DM, of whom 47% were previously undiagnosed, 5.5% had IFG, and 10.9% IGT. When we further categorized the HFDP post hoc using modified WHO 2013 GDM criteria, progression to T2DM was 83% and 31% in the DIP and GDM categories, respectively. Factors associated with risk of T2DM were fasting and OGTT 2-hour glucose concentration at HFDP diagnosis, oral hypoglycaemic and insulin treatment during HFDP, primary school education, BMI, and waist and hip circumferences at follow-up.

A key consideration for this study is the impact of GDM definition changes on the progression to type 2 diabetes. Recommendations of the IADPSG [8], based on findings from the HAPO Study were adopted by WHO in 2013 [10], and since then, most regional bodies have moved towards adopting WHO guidelines. Consequently, most studies published before 2013 used GDM definitions, such as the 1999 WHO guidelines on the diagnosis of GDM, which included both women with GDM and women with DIP and may therefore have overestimated the progression to T2DM proportion. In S2 Table, we have listed studies that investigated progression to T2DM in the medium- to long-term postpartum period, published during the period of 2000 to 2019, and the proportion of women who progressed to T2DM from each study. Most of these studies [32–46] used either WHO 1999 guidelines or other criteria, whereas only 4 studies used the IADPSG or equivalent criteria [11,21,22,47] for the diagnosis of GDM. Therefore, any comparisons of our findings with published data will need consideration of the heterogeneity of HFDP and GDM definitions.

In the South African context, the T2DM prevalence in our study population is 4 times higher than that of South African women overall (11%) [48] and higher than the T2DM prevalence in black women aged between 25 and 74 years (13.8%) [49] or women of mixed ancestry aged over 30 years (28.2%) [50] in Cape Town. Clearly, South African women with a history of HFDP are a vulnerable population and require intervention to delay or prevent progression to T2DM. The high proportion of women who progressed to T2DM (48%) could be explained partly by the possibility that, for some of the women, their glucose never returned to normality because they were not evaluated 6 weeks post the index pregnancy. This highlights the need for postpartum screening in these women. Global data indicates that postpartum screening is between 24% and 58% [51], whereas, in South Africa, less than 30% attend the recommended 6 weeks postpartum OGTT [24]. Several barriers from both the health system and patient perspectives hinder the 6-week postpartum screening. The South African health system is overburdened [52], and postpartum screening for diabetes at 6 weeks using the recommended 2-hour OGTT would add significantly to the burden. Although there are no South African data on barriers to postpartum screening, other studies have shown that the inconvenience of the OGTT and lack of time are the main reasons women do not attend the postpartum screening [51]. There is ongoing debate on the utility of either fasting glucose only or the HbA_1C_ for the postpartum screening [51]. Research is required to establish the optimum method to replace the OGTT, and for the HbA1C, both optimum timing for screening as well as cut-offs for the diagnosis of type 2 diabetes in African women. In the Western Cape, after delivery, the women must attend diabetes screening at a separate clinic while taking their offspring to a well-baby clinic for vaccination and follow-up, which may result in most women prioritizing the baby’s care over theirs. Studies investigating the barriers to postpartum screening as well as optimum screening methods in South African women are needed.

We found very different proportions of women who progressed to T2DM between the GDM and DIP groups. The proportion of women who progressed to T2DM in the GDM group was 31% when we recategorized the women using modified WHO 2013 criteria. The high proportion of women who progressed to T2DM in the DIP group (83% on OGTT alone but 96% on both HBA_1C_ and OGTT) suggests that they may possibly have had T2DM before the pregnancy. However, they clearly had more severe glucose intolerance during the pregnancy compared with the GDM group. When HbA_1C_ assessment was added, only 3 (4%) women in the DIP group did not progress to T2DM. Further analysis of the DIP group showed discordance between the OGTT results and HbA_1C_; 2 of the 7 participants with either impaired glucose intolerance or IFG had HbA_1C_ levels above 6.5% (7.2% and 8.6%), whereas 3 out of 6 participants with normal GTT had HbA_1C_ levels of at least 6.5% (6.5%, 6.5%, and 6.6%). The remaining 3 participants with normal GTT had HbA_1C_ levels below 6.5%. Our data, although in a small sample of women with DIP, suggests the need for more structured follow-up for assessment for T2DM after the pregnancy.

Comparisons of our findings with other studies that have investigated progression to T2DM is complicated by several issues. Firstly, there are no African studies that have investigated progression to T2DM post HFDP; the HAPO studies did not include an African cohort. Secondly, and more importantly, the heterogeneous definitions used for HFDP and GDM in the published studies (S2 Table) make it difficult to compare proportions of women who progressed to T2DM. Lastly, comparisons with published data are further complicated by the different study designs and different lengths of follow-up from the different studies. The proportion of women who progressed to T2DM of 31% in our GDM group, classified according to a modified WHO 2013 criteria, is somewhat high, compared with the 4 studies [11, 21, 22, 47] that used either the IADPSG or other criteria almost similar to it. This may be due to the cut-offs we used for GDM. Our study population is slightly different in that we did not include women with fasting glucose values between 5.1 and 5.5mmol/L, whereas we included women with OGTT 2-hour glucose values between 7.8 and 8.4mmol/L. The women in our study population, in terms of diagnostic glucose values, would have been almost similar to those included by Chamberlain and colleagues [21], in which widely different proportions of progression to T2DM for indigenous (25.5%) and nonindigenous women (5.7%) at 5 years post partum were reported. The proportion of women who progressed in the indigenous women was fairly similar to our study. A study from India by Gupta and colleagues [22] in women diagnosed using the IADPSG GDM criteria found that 25% and 42% of women aged 20 to 29 years and 30 to 39 years, respectively, progressed to T2DM in 5 years. The remaining 2 prospective cohort studies that used the IADPSG criteria for the diagnosis of GDM had follow-up periods that are very different from ours. In Japan, Inoue and colleagues [47] found that 22% progressed to T2DM 2 years post GDM, whereas 7.9% progression was observed in the HAPO Study [11] after a median follow-up of 11.4 years. It seems that progression to T2DM is heterogeneous, even when similar criteria for GDM diagnosis are used.

Identifying risk factors for the risk of progression to T2DM is a necessary step when designing interventions to delay or prevent T2DM. The risk factors for progression in our study are largely similar to findings from previous studies: fasting [53] and 2-hour OGTT glucose concentration [47,54] at HFDP diagnosis, and, at follow-up, BMI, waist and hip circumferences [39,54,55]. Insulin and oral hypoglycaemic treatment during HFDP are an indicator of the severity of HFDP and, in our study, 77% of women who had insulin treatment were classified as DIP. Of the women with dyslipidemia, 66% were already on treatment for diabetes at the time of follow-up, and it is well known that uncontrolled diabetes is associated with higher triglyceride and lower high density lipoprotein cholesterol (HDL-C) levels [56], and the participants with an established diabetes diagnosis were more likely to have been screened for dyslipidemia as part of standard care [57]. Although no other long term follow-up studies have shown a similar association between T2DM and education as ours, Gante [58] found an association between lower education and persistent postpartum glucose disorders in Portuguese women at 6 weeks follow-up. In our study and our setting, education is a good indicator of socioeconomic status, and therefore may be associated with an inability to access healthier lifestyle options such as better diets. Women with lower education may also not be able to access information on reducing T2DM risk after HFDP; therefore special interventions may be required for this group.

Preventing T2DM can be achieved through either population-wide approaches, such as the sugar tax, or interventions targeted at high-risk populations. The latter requires the screening and identification of high-risk individuals and offering interventions. Various diabetes prevention programs in both high-income and low-to-medium-income countries [46–49] have shown that lifestyle interventions can reduce the risk of T2DM in high-risk populations, such as people with IGT, although screening for IGT in a population can be expensive and difficult. Our study highlights the notion that women with a history of GDM are an obvious and easily accessible target for prevention because they are diagnosed as part of routine care in the health system. An added benefit of this approach is that by targeting these women, there is a real chance of decreasing the risk of intergenerational transmission of T2DM to the offspring. In South Africa, there are increasing calls for universal screening for GDM [18,19], which is costly and adds to the workload of health workers compared with the risk-factor–based screening, which leaves a substantial proportion of women with GDM unscreened. Regardless of the screening approach used, research on the efficacy or effectiveness of lifestyle interventions in preventing or delaying progression to T2DM in women post HFDP in South Africa would provide much-needed data.

Our study has several limitations. We were only able to follow-up with 44.2% of women after 5 to 6 years, comparable with other studies [46,47,53] and partly explained by a highly mobile population in the Western Cape, where in-and-out migration is common [59]. The women who participated were more likely to book 2 weeks early (15 versus 17 weeks), had a higher BMI at booking by 2 units (34.6 versus 32.7 kgm^2^), and had lower 2-hour OGTT blood glucose at HFDP diagnosis (9.0 versus 12.0 mmol/L) compared with the women who were lost to follow-up and therefore not completely representative of our study population. Due to the design, our study did not follow up women until diabetes developed, and therefore we do not have time to development of diabetes, as well as being unable to establish temporality for any of the risk factors we identified. The lack of a control group of women with normoglycemic pregnancies at the same time as our sample is a further limitation. However, when compared with recent T2DM prevalence in similar aged women in the Western Cape, our data indicate a high T2DM prevalence in women with a history of HFDP. More robust studies, with control groups, may be needed to further investigate our findings.

## Conclusion

Almost half of the women with a history of HFDP progress to T2DM within 5 to 6 years, with almost half of them undiagnosed, in Cape Town, South Africa. There is a need for postpartum screening and interventions to reduce the risk of progression.

## Supporting information

S1 STROBE Checklist STROBE checklist for the study. STROBE, Strengthening the Reporting of Observational Studies in Epidemiology.
(PDF)

S1 Doc Research protocol for the study. (PDF)

S2 Doc Questionnaire for the study. (PDF)

S1 Table Comparison of participants followed up 5 to 6 years later and the participants lost to follow-up. (DOCX)

S2 Table Progression to T2DM after HFDP—Studies published from 2000 to 2019. HFDP, hyperglycemia first detected in pregnancy; T2DM, type 2 diabetes mellitus.
(DOCX)

S1 Fig Box plot comparing fasting blood glucose at HFDP diagnosis by diabetes status at follow-up. (TIF)

S2 Fig Box plot comparing OGTT 2-hour blood glucose at HFDP diagnosis by diabetes status at follow-up. HFDP, hyperglycemia first detected in pregnancy; OGTT, oral glucose tolerance test.
(TIF)

S3 Fig Box plot comparing waist circumference at follow-up by diabetes status at follow-up. (TIF)

S4 Fig Box plot comparing hip circumference at follow-up by diabetes status at follow-up. (TIF)

S5 Fig Box plot comparing waist-hip ratio at follow-up by diabetes status at follow-up. (TIF)

S1 Data Study data. (XLSX)

## Abbreviations

ARV: anti-retroviral therapy
BMI: body mass index
CVD: cardiovascular disease
DIP: diabetes in pregnancy
FPG: fasting plasma glucose
GDM: gestational diabetes mellitus
GPAQ: global physical activity questionnaire
GSH: Groote Schuur Hospital
HAPO: Hyperglycaemia and Adverse Pregnancy Outcomes
HbA_1C_: glycated haemoglobin A_1c_
HDL: high density lipoprotein cholesterol
HFDP: hyperglycemia first detected in pregnancy
IADPSG: International Association of Diabetes and Pregnancy Study Group
IFG: impaired fasting glucose
IGT: impaired glucose tolerance
IQR: interquartile range
NICE: National Institute for Health and Care Excellence
OGTT: oral glucose tolerance test
OR: odds ratio
PA: physical activity
STROBE: Strengthening the Reporting of Observational Studies in Epidemiology
T2DM: type 2 diabetes mellitus
