# Supplementary material for: Progression to type 2 diabetes mellitus and associated risk factors after hyperglycemia first detected in pregnancy: A cross-sectional study in Cape Town, South Africa
Source: PLoS Med. 2019 Sep 9;16(9):e1002865. doi: 10.1371/journal.pmed.1002865 (PMC6733438; doi:10.1371/journal.pmed.1002865)
Supplement: S1 Doc — (PDF) [file pmed.1002865.s002.pdf]

S1 – PRO2D Research Protocol

Title: **The prevalence of type 2 diabetes mellitus and associated risk factors in women who had gestational diabetes mellitus 5 years previously, in South Africa**

Tawanda Chivese[1,2], Professor Shane Norris[2], Professor Naomi Levitt[1,3]

1. Chronic Disease Initiative for Africa, Department of Medicine, University of Cape Town

2. MRC/Wits Developmental Pathways for Health Research Unit, Department of Pediatrics, University of the Witwatersrand, South Africa.

3. Division of Endocrinology, Department of Medicine, Faculty of Medicine and Health Sciences, University of Cape Town.

Table of Contents

Abstract iii

Key Words ....

iii Abbreviations

.... iii

1. Background 1

1.1.1 Gestational diabetes and prevalence of type 2 diabetes mellitus 1

1.2 Rationale and Motivation 2

1.2 Aim and Objectives 2

1.3.1 Objectives 2

Methods 2

2.1 Research Design 2

2.2 Study Setting 2

2.3 Study population and sampling procedures 3

2.3.1 Inclusion Criteria 3

2.3.2 Exclusion criteria 3

2.4 Measurements 3

2.5 Study procedures 3

2.7 Questionnaire 4

2.8 Anthropometry and blood pressure
.... 4

2.9 Oral Glucose Tolerance Test and HbA1c 4

2.11 Sample Size 5

2.13 Data management and analysis 5

2.13.1 Data capturing 5

2.13.2 Statistical data analysis 6

2.14 Ethical considerations 6

1. Project Budget - Summary (see attached Appendix 1) 7
2. Research Timelines and Plan – Summary (see attached Appendix 2) 8
3. Dissemination of research results 8
4. References 8

Appendix 1: University of Cape Town Human Research Ethics Committee Approval.......... 12

Appendix 2: Groote Schuur Hospital Approval 13

Abstract

Background

Women with a history of gestational diabetes mellitus (GDM) are at high risk of progressing to type II diabetes mellitus (T2DM) and require frequent screening after delivery for early detection and intervention. In addition, their children may have an increased risk for overweight and obesity during their lifespan. Such data are not available in South Africa and the sub-Saharan African region, where the potential factors that may increase the risk of progression to T2DM as well as the metabolic syndrome may differ from risk factors in high income countries. These include high rates of food insecurity and obesity co-existing with undernutrition together with a potentially different genotype.

The overall objective of this study is to determine the prevalence of and risk factors for T2DM in postpartum women from low socioeconomic urban settings in South Africa who were diagnosed and treated for GDM 5 years ago in the public healthcare system.

Methods

The proposed cross-sectional study will take place at a referral centers (the Groote Schuur Hospital) over a period of 12 months. Women treated for GDM 5 years previously, identified from the existing registers, who fit the inclusion criteria will be contacted and invited to participate.

Each participant will be expected to participate for around 2 hours. Participants will undertake a 2-hour oral glucose tolerance test as well as a 30 – 45-minute interviewer administered questionnaire. Additional data will be collected through a review of medical records for all participants.

Key Words

Gestational diabetes, type II diabetes mellitus, glycated hemoglobin (HbA1c), metabolic syndrome, epidemiology, South Africa

Abbreviations

DM – diabetes mellitus

GDM – gestational diabetes mellitus

T2DM – type II diabetes mellitus
HbA1c – glycated hemoglobin

OGTT – oral glucose tolerance test

IDF – international diabetes federation

1. Background

Diabetes mellitus (DM) is one of the leading causes of morbidity and mortality in the world, with an estimated global prevalence of 9% in 2014 (1) and 1.5 million deaths directly related to DM in 2012 (2). At least 80% of DM related deaths occur in low- and middle-income countries (2). Around 90% of all DM cases are Type 2 diabetes mellitus (T2DM) (3). In South Africa, recent evidence shows that the prevalence of DM is increasing in urban dwelling South African populations (53% increase in prevalence during the period 1990 - 2012), including among women of child bearing age (4).The latest estimates are that the overall prevalence of diabetes in South Africa is 9% (5). The rising prevalence of non-communicable diseases like T2DM will add a considerable burden to an already struggling South African health system.

1.1.1 Gestational diabetes and prevalence of type 2 diabetes mellitus

Gestational diabetes mellitus (GDM) is a known risk factor for the development of T2DM (6). Initially GDM was defined as glucose intolerance first presenting in pregnancy with return to normal glucose tolerance post pregnancy (6). More recent definitions include both women with pre-existing diabetes first recognized in pregnancy and women who manifest glucose abnormalities for the first time during pregnancy. There is still no consensus on the mechanisms underlying the development of GDM, although greater insulin resistance in GDM women compared to matched pregnant controls during the third trimester of pregnancy has been reported by several authors (7-9), lending weight to the role played by insulin resistance in the pathophysiology of GDM.

Several studies have demonstrated that the prevalence of GDM varies in different populations (10-12), because this is directly related to the specific population prevalence of T2DM. Recent systematic reviews of the small number of available studies in Africa estimate GDM prevalence to be between 0 and 14% (13). In South Africa, evidence from an ongoing unpublished study (Soweto pilot study) estimates the GDM prevalence to be higher than 15%.

Systematic reviews, primarily based on data from high income countries have reported that women with previous GDM have a seven-fold risk of developing T2DM (6,14), increased risk of long term cardiovascular disease (15,16) and risk of adverse perinatal outcomes for the GDM offspring (15,16). Research from high income countries has also shown that the majority (around 50%) of the women who progress to T2DM after GDM do so within the first 5 years after the GDM pregnancy (17). In addition, the prevalence of T2DM in women with

previous GDM varies widely in different populations, Kim et al, for example, found that T2DM prevalence ranged from a low 3% in some populations to as high as 70% in other populations (17). There is no data on the proportion of women with previous GDM who progress to T2DM and associated risk factors in Sub-Saharan Africa where, unlike the highincome countries, there are high rates of food insecurity and obesity co existing with malnutrition together with a potentially different genotype.

1.2 Rationale and Motivation

Although evidence from systematic reviews suggests that GDM is a risk factor for T2DM, the extent of this risk is not known in Africa nor are the factors associated with the risk of development of T2DM. The T2DM disease burden in this high-risk population is not known in sub-Saharan Africa. The factors that may increase the risk of T2DM development in this vulnerable group may differ to those identified in high income countries. Any future interventions for the prevention of T2DM in women with a history of GDM need to be informed by evidence which is specific to the African context.

1.2 Aim and Objectives

The aim of the study is to determine the prevalence of and risk factors for T2DM in women who had GDM 5 years previously, in South Africa.

The aim will be achieved by the following objectives;

1.3.1 Objectives

1. To estimate the prevalence of T2DM in women who were diagnosed and received management for GDM 5-6 years previously by using a glucose tolerance test to identify women with T2DM.
2. To investigate the other subsequent factors for the development of T2DM in women who were diagnosed and received management for GDM 5-6 years previously.

Methods

2.1 Research Design

We will conduct a cross-sectional study to estimate the prevalence of T2DM and analyze possible risk factors in women with previous GDM.

2.2 Study Setting

The study will take place at a tertiary referral center, in the Western Cape Province (Groote Schuur Hospital).

2.3 Study population and sampling procedures

In the major South African cities, all women with GDM are managed at tertiary hospitals. Eligible participants who were managed for GDM during 2010 to 2011 at Groote Schuur

academic hospital will be identified from hospital registers and invited to participate in the study. The study involves women who had gestational diabetes 5 years ago and currently we have been calling them and asking them to come to Groote Schuur hospital for participation, in Cape Town. However, most of the contact numbers are now obsolete and we would like to amend the protocol to include that:

a. Home visits will be made where applicable, to recruit participants

b. Instead of the oral glucose tolerance test, a fasting blood glucose, fasting lipids as well as HbA1C will be made at community centers near the participants' locations in the case of participants who are unable to come to the Groote Schuur.

2.3.1 Inclusion Criteria

Adult women, aged over 18 years old, who were diagnosed and treated with GDM during the period 2010 – 2011.

2.3.2 Exclusion criteria

Women with known Type I or T2DM prior to the index pregnancy will be excluded from the study.

For the HbA1c test, women with the following will be excluded from the final analysis:

- Women who are being treated for chronic renal failure or
- Women who are being treated for iron deficiency anaemia or
- Women who are already on treatment for T2DM

2.4 Measurements

Diabetes as defined by the result of the 75g 2hour OGTT, as defined according to the WHO 2006 guidelines will be the main outcome. Other outcomes will include impaired fasting glucose and impaired glucose tolerance.

2.5 Study procedures

Women treated for GDM 5 years previously will identified from the existing registers, who fit the inclusion criteria will be contacted and invited to participate. Those who verbally agree to participate will be advised about the OGTT and the fasting requirements. The participation

will be roughly two and a half hours. Upon arrival at the study site, the study will be explained to the participants and they will be requested to sign a written informed consent. Once this is signed they will undergo the study procedures: blood samples for an HbA1c test, a 2-hour OGTT, a 30 – 45-minute interviewer will administer the questionnaire, anthropometry and blood pressure will be measured. A medical chart review will be undertaken for each participant after they have finished the OGTT and questionnaire.

2.6 Medical chart review

Clinical data pertinent to the index pregnancy will be collected via a review of the individual patient hospital records, using a structured data collection form. Data collected will include: maternal age gestational age and OGTT result at time of GDM diagnosis, insulin and oral hypoglycemic agent use during GDM, BMI at booking, infant birthweight (macrosomia or not – using birthweight of 4000g as cut off), and outcome of pregnancy (stillbirth and fetal complications), parity, previous GDM, and comorbidities such as pre-eclampsia, lipid levels if measured and diabetes therapy at time of discharge.

2.7 Questionnaire

The questionnaire will have:

- Demographic data section– data on age, level of education, marital status, area of residence, employment status and ethnicity. Ethnicity will be self-reported and care will be taken to include an “other” option for ethnicity to accommodate participants who may not be comfortable in disclose their ethnicity.
- Medical history – data on comorbidities e.g. hypertension, post GDM pregnancy breastfeeding, family history of Type 2DM, parity, self-reported postnatal DM followup and current medications including anti-retroviral therapy.
- Other variables – data on smoking history, alcohol use history, psychosocial health, diet and physical activity will be assessed using established methods (4).

2.8 Anthropometry and blood pressure

Height, weight, and waist and hip circumferences will be measured using standardized techniques (34).

2.9 Oral Glucose Tolerance Test and HbA1c

Participants will be given 75g anhydrous glucose in 250ml of water, in the morning after a 10-12 hour overnight fasting period (3). Venous blood samples will be taken at 0 minutes for glucose, lipogram and for HbA1c measurement, at 30 minutes and 120 minutes for glucose

and insulin. The bloods will be kept on ice, and sent to the laboratory for handling and measurement of the same day using commercially available test kits. T2DM will be diagnosed according to WHO guidelines (3). One additional blood sample (5ml each) will be taken from each participant, centrifuged and the serum stored in case some samples get spoiled or if confirmation is needed or an additional investigation relating to GDM is considered. The samples will be stored up to 5 years after the study and thereafter destroyed. Ethics approval will be sought if any additional investigations are performed. Any participants who are already on treatment for T2DM will not be required to do either the OGTT or the HbA1c.

2.11 Sample Size

Sample size for evaluating the prevalence of postnatal T2DM was calculated using the formula

$$\frac{p(1-p)z^2}{d^2}$$

With p = the hypothesized proportion of GDM women who have postnatal T2DM, z = 1.96 (corresponding to a 0.05 level of significance) and d = the precision to which the prevalence is to be estimated.

Most studies found a prevalence of T2DM during the first 5 years after GDM diagnosis between 20% and 50% (14).

The total number of women treated for GDM at the hospital during the period 1 September 2010 to 31 August 2011 was 498. Using 498 as the total population, a hypothesized prevalence of 40% and aiming to estimate the prevalence of T2DM to within 5 percentage points (d = 0.05), the study would require a sample size of 213.

Since the population is finite, we will endeavor to recruit all the 498 women, however, conservatively estimate that the study will be able to recruit 50% of these women, as our population is highly mobile and, apart from loss to follow-up and relocation, contact details in the hospital registers are not very accurate and some change their contact details.

2.13 Data management and analysis

2.13.1 Data capturing

All data will be captured on a pre-coded Microsoft Office Access Database (Microsoft Corporation, 2013) by trained research assistants. Double data entry will be used. All data will be de-identified before capturing by using assigned participant study identities. Signed informed consent forms and questionnaires will be kept in secure locked cabinets.

2.13.2 Statistical data analysis

All statistical analysis will be carried out using Stata 14 (37). For all analysis, the level of significance will be set at 0.05, 2-sided p-values and 95% Confidence Interval (95%CIs) will be reported. Crude T2DM prevalence will be calculated taking into consideration the sample design and the realized sample (4). For categorical variables frequencies and proportions will be reported, while bar graphs and pie chart will be used to present data where appropriate. The chi squared test, Fischer's Exact (small frequencies) and McNemar's chi squared Test (paired data) will be used to compare categories.

All continuous data will be tested for Normality using the Shapiro Wilks test and histogram plots. Means and standard deviations will be presented for variables that are normally distributed while medians and IQRs will be reported for variables that do not follow the Normal Distribution. Box and whisker plots, histograms, scatter plots and line graphs will be used to present data where appropriate. The t-tests and ANOVA (more than two groups) will be used to test to compare continuous outcomes between groups if data are normally distributed. For data that are not normally distributed, comparisons will be tested using Mann U Whitney, Wilcoxon sum rank (2 independent groups), Wilcoxon rank tests (paired data) as well as the Kruskal-Wallis for more than two groups.

Multivariate logistic regression, will be used to evaluate risk factors. Odds Ratios (ORs), their 95%CIs and p-values will be reported. Variables with p-values below 0.1 in the univariate analysis will be considered for the multivariate logistic regression. Additional analysis will include subgroup analysis for different ethnicities, should ethnicity prove to be an independent predictor of risk of T2DM.

2.14 Ethical considerations

This study will be performed in accordance with the principles with the Declaration of Helsinki (38).

The participants will be given advice on good food choices and physical activity at the conclusion of the study. They will also receive a "health card" with the results of all their anthropometric measurements and blood tests.

If any medical abnormalities (such as undiagnosed diabetes or hypertension) are identified during data collection the participants will be referred to their nearest day hospital for followup and management.

Permission to conduct the research will be sought from the Department of Health, the Groote Schuur, the Tygerberg Hospital and the Chris Hani Baragwanath Hospitals.

The study has received ethics clearance from the Human Research Ethics Committees of the University of Cape Town (Ref: 656/2015, Appendix 1) as well as permission to conduct research at the Groote Schuur Hospital (Appendix 2) in Cape Town.

The study will involve drawing blood samples from the forearms. Some of the potential risks in the blood collection include infection, delayed healing, bruising and some physical pain. The blood samples will be drawn using experienced nurses to minimize the risks.

All data will be de-identified during data capturing while the blood samples will be deidentified during alliquoting. The stored samples will not be used for any purpose other than the aims of the study. Any further use will only be carried out after ethical clearance from the UCT Human Research Ethics Committee.

All participants will go through the informed consent process which will involve an explanation of the study, the risks and benefits, the nature of their participation as well as their right to refuse to participate as well as stop their participation at any stage during or after the research.

The participants will receive a R100 to compensate for their travel expenses.

The lead investigators, the PhD student and the study coordinators are trained in good clinical practice (GCP). GCP principles will be adhered to during all stages of the conduct and reporting of the research.

1. **Project Budget - Summary** (see attached Appendix 1)

**SUMMARY**	
Personnel	158000.00
Travel	24400.00
Equipment	18000.00
Materials & Supplies	10000.00
**Laboratory supplies and analysis**	147,200.00
Other Direct Costs	158000.00
**TOTAL**	**516000.00**
**sample size 400 (200 per site)*	

2. **Research Timelines and Plan –** Summary (see attached Appendix 2)

This proposed schedule will be implemented when ethics approval, permissions and the relevant funding are obtained.

Activity	Dates	Duration (months)	Principal investigators	Students	Other personnel	Costs
Research Proposal development and ethics	Month 1 –2	2	Profs Levitt & Prof Norris	1 x PhD student		Personnel salaries
Pilot Study	Month 3	1 month	Profs Levitt & Prof Norris	1 x PhD student	2 x research nurses, 2 x field workers	Personnel salaries consumables; lab tests; patient transport, refreshment and time remuneration, airtime
Data collection	Months 4 - 10	6 months	Profs Levitt & Prof Norris	1 x PhD student	2 x research nurses, 2 x field workers	Personnel salaries airtime, OGTT &HbA1c tests, patient reimbursement, stationary costs.
Data analysis, write up, presentation and publication	Months 11 - 14	4 months	Profs Levitt & Prof Norris	1 x PhD student		Personnel salaries

3. Dissemination of research results

The research results will be published in international and regional peer reviewed journals. The results will also be presented at conferences at academic conferences as well as conferences with key stakeholders and policymakers.

4. References

(1) World Health Organization. Global status report on noncommunicable diseases 2014.

(2) World Health Organization. Global Health Estimates: Deaths by Cause, Age, Sex and Country, 2000-2012. Geneva, WHO 2014.

(3) World Health Organization. Definition, Diagnosis and Classification of Diabetes Mellitus and its Complications: Report of a WHO Consultation. Geneva: World Health Org 1999.

(4) Peer N, Steyn K, Lombard C, Lambert E, Vythilingum B, Levitt N. Rising diabetes prevalence among urban-dwelling black South Africans. PloS one 2011;7(9):e43336e43336.

(5) Bertram MY, Jaswal AV, Van Wyk VP, Levitt NS, Hofman KJ. The non-fatal disease burden caused by type 2 diabetes in South Africa, 2009. Global health action 2013;6.

(6) Bellamy L, Casas J, Hingorani AD, Williams D. Type 2 diabetes mellitus after gestational diabetes: a systematic review and meta-analysis. The Lancet 2009;373(9677):1773-1779.

(7) Buchanan TA. Pancreatic B-cell defects in gestational diabetes: implications for the pathogenesis and prevention of type 2 diabetes. J Clin Endocrinol Metab 2001 Mar;86(3):989-993.

(8) Homko C, Sivan E, Chen X, Reece E, Boden G. Insulin Secretion during and after Pregnancy in Patients with Gestational Diabetes Mellitus 1. The Journal of Clinical Endocrinology & Metabolism 2001;86(2):568-573.

(9) Kautzky-Willer A, Prager R, Waldhausl W, Pacini G, Thomaseth K, Wagner OF, et al. Pronounced insulin resistance and inadequate beta-cell secretion characterize lean gestational diabetes during and after pregnancy. Diabetes Care 1997 Nov;20(11):17171723.

(10) Hedderson MM, Darbinian JA, Ferrara A. Disparities in the risk of gestational diabetes by race-ethnicity and country of birth. Paediatr Perinat Epidemiol 2010;24(5):441-448.

(11) Dooley SL, Metzger BE, Cho NH. Gestational diabetes mellitus: influence of race on disease prevalence and perinatal outcome in a US population. Diabetes 1991;40(Supplement 2):25-29.

(12) Metzger BE, Buchanan TA, Coustan DR, de Leiva A, Dunger DB, Hadden DR, et al. Summary and recommendations of the Fifth International Workshop-Conference on Gestational Diabetes Mellitus. Diabetes Care 2007 Jul;30 Suppl 2:S251-60.

(13) Macaulay S, Dunger DB, Norris SA. Gestational diabetes mellitus in Africa: a systematic review. PloS one 2014;9(6):e97871.

(14) Kim C, Newton KM, Knopp RH. Gestational diabetes and the incidence of type 2 diabetes: a systematic review. Diabetes Care 2002 Oct;25(10):1862-1868.

(15) Schmidt MI, Duncan BB, Reichelt AJ, Branchtein L, Matos MC, Costa e Forti A, et al. Gestational diabetes mellitus diagnosed with a 2-h 75-g oral glucose tolerance test and adverse pregnancy outcomes. Diabetes Care 2001 Jul;24(7):1151-1155.

(16) Peters RK, Xiang A, Kjos S, Buchanan T. Long-term diabetogenic effect of single pregnancy in women with previous gestational diabetes mellitus. The Lancet 1996;347(8996):227-230.

(17) Kim C, Newton KM, Knopp RH. Gestational diabetes and the incidence of type 2 diabetes: a systematic review. Diabetes Care 2002 Oct;25(10):1862-1868.

(18) Kassi E, Pervanidou P, Kaltsas G, Chrousos G. Metabolic syndrome: definitions and controversies. BMC Med 2011 May 5;9:48-7015-9-48.

(19) Xu Y, Shen S, Sun L, Yang H, Jin B, Cao X. Metabolic syndrome risk after gestational diabetes: a systematic review and meta-analysis. PloS one 2014;9(1).

(20) Jennings CL, Lambert EV, Collins M, Joffe Y, Levitt NS, Goedecke JH. Determinants of Insulin-resistant Phenotypes in Normal-weight and Obese Black African Women. Obesity 2008;16(7):1602-1609.

(21) Goedecke JH, Levitt NS, Lambert EV, Utzschneider KM, Faulenbach MV, Dave JA, et al. Differential effects of abdominal adipose tissue distribution on insulin sensitivity in black and white South African women. Obesity 2009;17(8):1506-1512.

(22) Lindsay RS, Nelson SM, Walker JD, Greene SA, Milne G, Sattar N, et al. Programming of adiposity in offspring of mothers with type 1 diabetes at age 7 years. Diabetes Care 2010 May;33(5):1080-1085.

(23) Clausen TD, Mathiesen ER, Hansen T, Pedersen O, Jensen DM, Lauenborg J, et al. Overweight and the metabolic syndrome in adult offspring of women with diet-treated gestational diabetes mellitus or type 1 diabetes. The Journal of Clinical Endocrinology & Metabolism 2009;94(7):2464-2470.

(24) Clausen TD, Mathiesen ER, Hansen T, Pedersen O, Jensen DM, Lauenborg J, et al. High prevalence of type 2 diabetes and pre-diabetes in adult offspring of women with gestational diabetes mellitus or type 1 diabetes: the role of intrauterine hyperglycemia. Diabetes Care 2008 Feb;31(2):340-346.

(25) Boerschmann H, Pfluger M, Henneberger L, Ziegler AG, Hummel S. Prevalence and predictors of overweight and insulin resistance in offspring of mothers with gestational diabetes mellitus. Diabetes Care 2010 Aug;33(8):1845-1849.

(26) Rijpert M, Evers IM, de Valk HW, de Vroede MA, Tersteeg-Kamperman M, Heijnen CJ, et al. Cardiovascular and metabolic outcome in 6–8year old offspring of women with type 1 diabetes with near-optimal glycaemic control during pregnancy. Early Hum Dev 2011;87(1):49-54.

(27) Stuart A, Amer-Wåhlin I, Persson J, Källen K. Long-term cardiovascular risk in relation to birth weight and exposure to maternal diabetes mellitus. Int J Cardiol 2013;168(3):26532657.

(28) Simmons D, McElduff A, McIntyre HD, Elrishi M. Gestational diabetes mellitus: NICE for the U.S.? A comparison of the American Diabetes Association and the American College of Obstetricians and Gynecologists guidelines with the U.K. National Institute for Health and Clinical Excellence guidelines. Diabetes Care 2010 Jan;33(1):34-37.

(29) Tovar A, Chasan-Taber L, Eggleston E, Oken E. Postpartum screening for diabetes among women with a history of gestational diabetes mellitus. Prev Chronic Dis 2011 Nov;8(6):A124.

(30) Venkataraman H, Sattar N, Saravanan P. Postnatal testing following gestational diabetes: time to replace the oral glucose tolerance test? The Lancet Diabetes & Endocrinology 2015.

(31) Saudek CD, Herman WH, Sacks DB, Bergenstal RM, Edelman D, Davidson MB. A new look at screening and diagnosing diabetes mellitus. The Journal of Clinical Endocrinology & Metabolism 2013.

(32) World Health Organization. Use of glycated haemoglobin (HbA1c) in diagnosis of diabetes mellitus: abbreviated report of a WHO consultation. 2011.

(33) Alberti KGM, Zimmet P, Shaw J, IDF Epidemiology Task Force Consensus Group. The metabolic syndrome—a new worldwide definition. The Lancet 2005;366(9491):1059-1062.

(34) Alberti K, Zimmet P, Shaw J. Metabolic syndrome—a new world-wide definition. A consensus statement from the international diabetes federation. Diabetic Med 2006;23(5):469-480.

(35) McLellan J, Barrow B, Levy J, Hammersley M, Hattersley A, Gillmer M, et al. Prevalence of diabetes mellitus and impaired glucose tolerance in parents of women with gestational diabetes. Diabetologia 1995;38(6):693-698.

(36) Bennett C, Guo M, Dharmage S. HbA1c as a screening tool for detection of type 2 diabetes: a systematic review. Diabetic Med 2007;24(4):333-343.

(37) Stata Corporation. College Station (TX). Stata Press 2014.

(38) Helsinki W. Do. World medical association declaration of Helsinki. Fortaleza, Brazil 2013.

Appendix 1: University of Cape Town Human Research Ethics Committee Approval

UNIVERSITY OF CAPE TOWN
Faculty of Health Sciences
Human Research Ethics Committee

Room E52-24 Old Main Building
Groote Schuur Hospital
Observatory 7925
Telephone [021] 406 6338 • **Facsimile** [021] 406 6411
Email: sumayah.ariefdien@uct.ac.za
Website: www.health.uct.ac.za/fhs/research/humanethics/forms

27 November 2015

HREC REF: 656/2015

Prof N Levitt
Division of Endocrinology
J-47
OMB

Dear Prof Levitt

PROJECT TITLE: THE PREVALENCE OF TYPE 2 DIABETES MELLITUS AND ASSOCIATED RISK FACTORS 5 YEARS AFTER GESTATIONAL DIABTES MELLITUS IN SOUTH AFRICA (PhD candidate-T Chivese)

Thank you for your response letter dated 23 November 2015, addressing the issues raised by the Human Research Ethics Committee (HREC).

It is a pleasure to inform you that the HREC has formally approved the above-mentioned study.

Approval is granted for one year until the 30th November 2016.

Please submit a progress form, using the standardised Annual Report Form if the study continues beyond the approval period. Please submit a Standard Closure form if the study is completed within the approval period.
(Forms can be found on our website: www.health.uct.ac.za/fhs/research/humanethics/forms)

We acknowledge that the following student:-Tawanda Chivese is also involved in this project.

Please quote the HREC reference no in all your correspondence.

Please note that the ongoing ethical conduct of the study remains the responsibility of the principal investigator.

Yours sincerely

PROFESSOR M BLOCKMAN
CHAIRPERSON, FHS HUMAN RESEARCH ETHICS COMMITTEE
Federal Wide Assurance Number: FWA00001637.
Institutional Review Board (IRB) number: IRB00001938

Hrec/ref:656/2015

Appendix 2: Groote Schuur Hospital Approval

Western Cape Government
Health

GROOTE SCHUUR HOSPITAL
Enquiries: Dr Bernadette Eick
E-mail : *Bernadette.Eick@westerncape.gov.za*

Professor N. Levitt
Division of Endocrinology
Department of Medicine
J47 – Old Main Building

E-mail: Naomi.Levitt@uct.ac.za / tchivese@gmail.com

Dear Professor Levitt

RESEARCH PROJECT: The Prevelance of Type 2 Diabetes Mellitus & Associated Risk Factors 5yrs After Gestational Diabetes Mellitus In South Africa (PhD Candidate T Chivese)

Your recent letter to the hospital refers.

You are hereby granted permission to proceed with your research and is valid until 30 November 2016.

Please note the following:

a) Your research may not interfere with normal patient care.
b) Hospital staff may not be asked to assist with the research.
c) No hospital consumables and stationary may be used.
d) **No patient folders may be removed from the premises or be inaccessible.**
e) Please introduce yourself to the person in charge of an area before commencing.
f) Please discuss the study with the HOD before commencing.
g) Please provide the research assistant/field worker with a copy of this letter as verification of approval.
h) Confidentiality must be maintained at all times.
i) Should you require additional research time beyond the stipulated expiry date, please apply for an extension.
j) Once research is complete, please submit a copy of the publication or report.

I would like to wish you every success with the project.

Yours sincerely

DR BERNADETTE EICK
CHIEF OPERATIONAL OFFICER
Date: 29th January 2016

C.C. Mr. L. Naidoo, Professor E. Weimann

G46 Management Suite, Old Main Building,
Observatory 7925

Private Bag X,
Observatory, 7935

Tel: +27 21 404 6288 fax: +27 21 404 6125

www.capegateway.go.v.za
