# Supplementary material for: Progression to type 2 diabetes mellitus and associated risk factors after hyperglycemia first detected in pregnancy: A cross-sectional study in Cape Town, South Africa
Source: PLoS Med. 2019 Sep 9;16(9):e1002865. doi: 10.1371/journal.pmed.1002865 (PMC6733438; doi:10.1371/journal.pmed.1002865)
Supplement: S2 Doc — (PDF) [file pmed.1002865.s003.pdf]

Participant Number

MRC/Wits Developmental Pathways for Health Research Unit

PRO2D - The prevalence of Type 2 diabetes mellitus and associated risk factors 5 years after gestational diabetes mellitus in South Africa

QUESTIONNAIRE

Tawanda Chivese, Professor Shane Norris, Professor Naomi Levitt

SECTION 1: GENERAL INFORMATION

Participant study number: ………………………………………………

Interviewer's name………………………………………………

Participant suburb of residence………………………………………………………………

Consent has been read and obtained? Yes, if yes continue

No, if no, STOP

Telephone…………………………..

2nd contact person's details:

Relationship…..……………………………..…………………………..……………………..………

Telephone ……………………………………………………………………………………….

Date of interview:

D D M M Y Y Y Y

Age of participant at last birthday ………………….……………………………

Participant date of birth:

D D M M Y Y Y Y

Participant Number

SECTION 2: SOCIO-DEMOGRAPHIC INFORMATION

	QUESTIONS AND FILTERS	CODING CATEGORIES	
2A	What is the highest level of education that you have achieved?		
		Never went to school	1
		Grade 1 to 7 (Primary school)	2
		Grade 8 to 10	3
		Matric	4
		Tertiary / Diploma	5
2B	Are you …	Employed, salaried	1
		Self-employed	2
		Unemployed	3
		A full-time homemaker	4
		A pensioner	5
		On a disability grant	6
		A student	7
2C	Do you own a house?	Yes …….. 1	No … 0
2D	What type of housing do you live in?	Built formal unit	1
		Informal shack / shelter/hostel/other	2
2E	How many rooms does your house have? (don't include bathroom & kitchen if separate)	……………………….	
2F	How many people, older than 18 years old, are living with you in your house?	………………………………..	
2G	Does your household have:	YES	NO
	Electricity?	1	0
	A radio?	1	0
	A television	1	0
	A telephone	1	0
	A refrigerator?	1	0
	A personal computer (PC)?	1	0
	A washing machine?	1	0
	Access to tap water:	1	0
	Tap in house	1	0

	QUESTIONS AND FILTERS	CODING CATEGORIES	
	Tap outside house	1	0
	Shared tap (4 houses)	1	0
	Communal tap (5 or more houses)	1	0
	A toilet	1	0
	A motor car	1	0
	A bicycle (adults)	1	0
2H	What ethnicity do you identify yourself as?		
		Black African	1
		Coloured	2
		White	3
		Indian/Asian	4
		Other	5
2I	Are you…….	Single	1
		Married (civil)	2
		Widowed/divorced	3
		Other	4
	If you answered other, please specify	………………………………………	
2M	When were you last tested for diabetes?	Months (……………………..)	
2N	What was the result of the test, if you tested for diabetes?	Diabetes ……….1<br>No diabetes …….2<br>Can't remember ……3<br>Not applicable ……..4	

SECTION 3: SELF-REPORTED REPRODUCTIVE HISTORY

**In this section I am going to ask you about your health as a mother**		
3A	Would you say your health is poor, average, good, or very good/excellent?	Poor .... 1<br>Average .... 2<br>Good....................... 3<br>Very good/excellent. 4

Participant Number

3B	Do you personally think that you are underweight, normal weight or overweight?	Underweight............1<br>Normal weight..........2<br>Overweight..............3<br>Don't know 9
3C	How many times have you been pregnant?	………………………
3D	Have you ever had any miscarriages?	Yes ………. 1<br>No ……… 0
3E	How many children do you have?<br><br>Please list the children and their details below, in 3F	……………………

3F	Name of child	Which year was the child born?	Did you breastfeed this child? (yes/no)	If you breastfed, how long did you breastfeed this child	Did you have diabetes during the pregnancy? (yes/no)	If you had diabetes, how were you treated?

3G	**Before your pregnancy in 2010/2011,** were you ever told by a doctor or a nurse that you had diabetes?	Yes……………1<br>No………………0
3H	If you answered yes to question 3G, were you being treated for diabetes (**before your pregnancy in 2010/2011**)	Yes………………1<br>No………………..0<br>Not applicable….5
3I	If you were being treated for diabetes **before your pregnancy in 2010/2011**, what treatment were you on?	Insulin only……1<br>Orals pills only…2<br>Insulin & oral pills…3<br>Diet………………..4<br>Not applicable…….5
3J	**After your pregnancy in 2010/2011, w**ere you given a referral letter to have your blood sugar checked**?**	Yes………………1<br>No………………0
3K	Did you go to have your blood sugar checked (for diabetes), **after you baby was born in 2010/2011**?	Yes………………1<br>No……………0

3L	If you went to be checked for diabetes, how many months **after your pregnancy in 2010/2011** did you go?	………………..
3M	If you went to be checked for diabetes **after your pregnancy in 2010/2011**, what test did you have?	Oral Glucose Tolerance Test ….1<br><br>Fingerprick …….2<br><br>Other……………...3<br><br>Don't remember ….4<br><br>Not applicable ….5
3N	If you went be checked for diabetes **after your pregnancy in 2010/2011**, what were the results?	Diabetes ……….1<br><br>No diabetes …….2<br><br>Can't remember ……3<br><br>Not applicable……4
3O	If you did not go to be checked for diabetes **after your pregnancy in 2010/2011**, why did you not go?<br><br>…………………………………………………………………………………………………	

SECTION 4: CHRONIC DISEASES AND MEDICATIONS

	**Since your pregnancy in 2011** has a **doctor** or **nurse** or **health worker** at a **clinic** or **hospital told you** that you have or have had any of the following conditions:	
4A	High Blood Pressure?	Yes..1<br>No..0<br>Don't know..9
4B	If you have high blood pressure, are you being treated?	Yes…………1<br>No…………..0<br>Not applicable …………99
4C	Heart attack or angina (chest pains)?	Yes..1<br>No..0<br>Don't know..9
4D	If you have had a heart attack, are you being treated?	Yes…………1<br>No…………0<br>Not applicable …………99

4E	Stroke?	Yes..1<br>No...0<br>Don't know...9
4F	If you have had a stroke, are you being treated?	Yes…………1<br>No…………..0<br>Not applicable …………99
4G	High blood cholesterol or fats in the blood?	Yes..1<br>No...0<br>Don't know...9
4H	If you have had high blood cholesterol, are you being treated?	Yes…………1<br>No…………..0<br>Not applicable …………99
4I	Diabetes	Yes..1<br>No...0<br>Don't know...9
4J	If you were told that you have diabetes, what treatment are you on?	Diet only……………0<br>Orals only………… 1<br>Insulin only ……….. 2<br>Orals and insulin…..3<br>No treatment ………. 4<br>Not applicable ……….5
4K	Kidney Disease	Yes..1<br>No...0<br>Don't know...9
4L	If you have had kidney disease, are you being treated?	Yes…………1<br>No…………..0<br>Not applicable …………99
4M	Cancer	Yes..1<br>No………………………………………………0<br>Don't know…………………………………..9
4N	If you have had cancer, are you being treated?	
4O	Chronic respiratory diseases, including asthma	Yes..1<br>No…………………………………………0<br>Don't know…………………………………9
4P	If you have had a chronic respiratory disease, are you being treated?	Yes…………1<br>No…………..0<br>Not applicable …………99

	QUESTIONS AND FILTERS	CODING CATEGORIES
	Now I want to ask you about any medication you take:	

	QUESTIONS AND FILTERS	CODING CATEGORIES
4Q	Do you use any medicine regularly or daily that a doctor or nurse has prescribed?	Yes ....1<br>No....................0<br>Don't know....................9

Record all the drugs mentioned

Drug	Date started	Date stopped	Still taking y/n	Reason for taking drug
**Diabetic medications (Insulin)**,				
**Diabetic medications (Oral hypoglycaemics),** e.g.				
Blood pressure medication				
Raised blood cholesterol medications e.g. Statins				
Others (Specify)				

SECTION 5: FAMILY MEDICAL HISTORY

**Now I would like to ask you about your family. Do you have a close blood relative (father, mother, brother, sister or child) who has ever been diagnosed by a doctor or nurse with any of the following conditions:**			
			**Who has it?**
5A	High Blood Pressure?	Yes ....1<br>No....................0<br>Don't know....................9	
5B	Heart attack or angina or chest pain when exerting himself/herself?	Yes ....1<br>No....................0<br>Don't know....................9	
5C	Stroke?	Yes ....1<br>No....................0<br>Don't know....................9	
5D	Diabetes?	Yes ....1<br>No....................0<br>Don't know....................9	

Participant Number

SECTION 6: ALCOHOL USE

**In this section I would like to ask whether you drink alcohol or not and how you drink it**		
6A	Have you **ever** consumed any alcohol such as beer, wine, spirits or sorghum beer?<br>**IF NO, GO TO SECTION 7**	Yes …………………..1<br>No……………………..0
6B	Have you consumed any alcohol within the **past 30 days**?	Yes …………………..1<br>No……………………..0
6C	If yes, during the past 30 days, **how frequently** have you had at least one standard alcoholic drink?	Daily 1<br>5-6 days per week 2<br>3-4 days per week 3<br>1-2 days per week 4<br>1-3 days per month 5<br>Less than once a month 6
6D	IF NO, have you stopped drinking due to health reasons, such as a negative impact on your health or on the advice of your doctor or other health worker?	Yes …………………..1<br>No…………………....0
6E	If you stopped drinking, when did you stop?	Year…………………….
6F	Have you ever felt that you should cut down on your drinking?	Yes……………………1<br>No……………………..0
6G	Have people annoyed you by criticizing your drinking?	Yes……………………..1<br>No………………………0
6H	Have you ever felt bad or guilty about your drinking?	Yes……………………1<br>No……………………..0
6I	Have you ever had a drink first thing in the morning to steady your nerves or get rid of a hangover?	Yes……………………1<br>No……………………..0

SECTION 7: SMOKING HISTORY

**Now, I would like to ask you whether you smoke tobacco products or not and if you do, how you do so.**			
7	Have you ever smoked cigarette products?<br><br>**IF NO, PLEASE GO TO QUESTION 7I**	Yes<br>1	No<br>0
7A	Do you currently smoke any tobacco products, such as cigarettes, cigars, or pipes?	Yes<br>1	No<br>0
7B	Do you currently smoke tobacco products **daily**?	Yes<br>1	No<br>0
7C	**How old** were you when you first **started** smoking daily?<br>Years old		

7D	If you do not remember how old you were, do you remember how long ago you started smoking daily?			
	1	WEEKS AGO		
	2	MONTHS AGO		
	3	YEARS AGO		
7E	On average, how many of the following items do you smoke each day? [NONE = 00]			
	1	Manufactured cigarettes?		
	2	Hand-rolled cigarettes?		
	3	Pipes full of tobacco?		
	4	Cigars/Cheroots/Cigarillos?		
7F	**In the past,** did you ever smoke daily?		Yes 1	No 0
7G	**How old** were you when you first **stopped** smoking daily? Years old			
	Don't remember/not sure = 77			
7H	If you do not remember how old you were, do you remember how long ago you stopped smoking daily?			
	1	Weeks Ago		
	2	Months Ago		
	3	Years Ago		
7I	Before you stopped smoking, how many of the following items did you smoke each day? [NONE = 00]			
	1	Manufactured cigarettes?		
	2	Hand-rolled cigarettes?		
	3	Pipes full of tobacco?		
	4	Cigars/Cheroots/Cigarillos?		
	**ASSESSING USE OF SMOKELESS TOBACCO**			
7J	Do you **currently use any** smokeless tobacco, such as snuff or chewing tobacco?		Yes 1	No 0
	**If No, go to question 7L**			

7K	Do you currently use smokeless tobacco **daily**?		Yes 1	No 0
	**If No, go to question 7L**			
7L	On average, how many times do you use each of the following items per day? [None = 00]			
	1	Snuff (by mouth)?		
	2	Snuff (by nose)?		
	3	Chewing tobacco?		
7M	**In the past,** did you ever use smokeless tobacco, such as snuff or chewing tobacco daily?		Yes 1	No 0
7N	During the past **30 days,** did someone smoke in your home?		Yes 1	No 0
7O	During the past **30 days,** did someone smoke in closed areas in your workplace (in the building, in a work area or a specific office)?		Yes 1	No 0

SECTION 8: PSYCHOSOCIAL HEALTH

We are now going to ask you questions relating to certain aspects of our lives. Some of the questions may seem the same but please bear with us because they are not exactly the same.

A) ORIENTATION TO LIFE QUESTIONNAIRE (SOC-13)

Below is a series of questions relating to various aspects of our lives. For each question, you SHOULD first select the response which most closely resembles how you feel. Then, on a scale from 1 to 7, you SHOULD select the number closest to that response which best describes how you feel. You can choose any number between 1 and 7. Please give only one answer to each question.

1. Do you have the feeling that you don't really care about what goes on around you?

very seldom or never ... very often

1 2 3 4 5 6 7

Participant Number

2. Has it happened in the past that you were surprised by the behaviour of people whom you thought you knew well?<br><br>never happened ... always happened<br>1 2 3 4 5 6 7
3. Has it happened that people whom you relied/depended on disappointed you?<br><br>never happened ... always happened<br>1 2 3 4 5 6 7
4. Until now your life has had:<br><br>no clear direction or purpose at all ... very clear direction and purpose<br>1 2 3 4 5 6 7
5. Do you have the feeling that you're being treated unfairly?<br><br>very often ... very seldom<br>1 2 3 4 5 6 7
6. Do you have the feeling that you are in an unfamiliar situation and don't know what to do?

Participant Number

very often very seldom<br>or never<br>1 2 3 4 5 6 7
7. Doing the things you do every day is:<br><br>a source of a source of frustration<br>deep pleasure and boredom<br>and satisfaction<br>1 2 3 4 5 6 7
8. Do you feel confused or have very mixed-up feelings and ideas?<br><br>very often very seldom<br>or never<br>1 2 3 4 5 6 7
9. Does it happen that you have feelings inside that you don't like or would rather not feel?<br><br>very often very seldom<br>or never<br>1 2 3 4 5 6 7

Participant Number

10. Many people--even those who are confident and successful--sometimes feel like losers in certain situations. How often have you felt this way in the past?<br><br>never ... very often<br>1 2 3 4 5 6 7
11. When something happened, have you generally found that:<br><br>you over-estimated or underestimated its importance? ... you saw things in the right perspective?<br>1 2 3 4 5 6 7
12. How often do you have the feeling that there's little meaning in the things you do in your daily life?<br><br>very often ... very seldom or never<br>1 2 3 4 5 6 7
13. Sometimes people have strong feelings that they cannot keep under control. How often do you have feelings that you're not sure you can keep under control?<br><br>very often ... very seldom or never<br>1 2 3 4 5 6 7

Participant Number

B) LOCUS OF CONTROL

To which extent do you agree or disagree with the following statements about your own life:

	Strongly disagree	Disagree	Neutral	Agree	Strongly agree
a) At work, I feel I have control over what happens in most situations.					
b) I feel what happens in my life is often determined by factors beyond my control.					
c) Over the next 5-10 years, I expect to have more positive than negative experiences.					
d) I often have the feeling I am being treated unfairly.					
e) In the past 10 years my life has been full of changes without my knowing what will happen next.					
f) I gave up trying to better my life a long time ago.					

C) LIFE EVENTS QUESTIONNAIRE

Have any of the following life events or problems happened to you during the last 6 months? How about more than 6 months ago? If so, please also rate the impact on you.

1. You yourself suffered a serious illness, injury or an assault. Yes/No. If no, go to next question.

Did this occur in past 6 months?	Yes / No
If yes, Impact:	None / Some / Significant

Participant Number

Did this occur more than 6 months ago?	Yes / No
If yes, Impact:	None / Some / Significant
2. A serious illness, injury or assault happened to a close relative. Yes/No. If no, go to next question.	
Did this occur in past 6 months?	Yes / No
If yes, Impact:	None / Some / Significant
Did this occur more than 6 months ago?	Yes / No
If yes, Impact:	None / Some / Significant
3. Your parent, child or spouse died. Yes/No. If no, go to next question.	
Did this occur in past 6 months?	Yes / No
If yes, Impact:	None / Some / Significant
Did this occur more than 6 months ago?	Yes / No
If yes, Impact:	None / Some / Significant
4. A close family friend or another relative (aunt, cousin, Grandparent) died. Yes/No.<br>If no, go to next question.	

Participant Number

Did this occur in past 6 months?	Yes / No
If yes, Impact:	None / Some / Significant
Did this occur more than 6 months ago?	Yes / No
If yes, Impact:	None / Some / Significant
5. You had a separation due to marital difficulties. Yes/No. If no, go to next question.	
Did this occur in past 6 months?	Yes / No
If yes, Impact:	None / Some / Significant
Did this occur more than 6 months ago?	Yes / No
If yes, Impact:	None / Some / Significant
6. You broke off a steady relationship. Yes/No. If no, go to next question.	
Did this occur in past 6 months?	Yes / No
If yes, Impact:	None / Some / Significant
Did this occur more than 6 months ago?	Yes / No

If yes, Impact:	None / Some / Significant
7. You had a serious problem with a close friend, neighbor or relative. Yes/No. If no, go to next question.	
Did this occur in past 6 months?	Yes / No
If yes, Impact:	None / Some / Significant
Did this occur more than 6 months ago?	Yes / No
If yes, Impact:	None / Some / Significant
8. You became unemployed or you were seeking work unsuccessfully for more than one month. Yes/No. If no, go to next question.	
Did this occur in past 6 months?	Yes / No
If yes, Impact:	None / Some / Significant
Did this occur more than 6 months ago?	Yes / No
If yes, Impact:	None / Some / Significant
9. You were fired from your job. Yes/No. If no, go to next question.	

Did this occur in past 6 months?	Yes / No
If yes, Impact:	None / Some / Significant
Did this occur more than 6 months ago?	Yes / No
If yes, Impact:	None / Some / Significant
10. You had a major financial crisis. Yes/No. If no, go to next question.	
Did this occur in past 6 months?	Yes / No
If yes, Impact:	None / Some / Significant
Did this occur more than 6 months ago?	Yes / No
If yes, Impact:	None / Some / Significant
11. You had problems with the police and a court appearance. Yes/No. If no, go to next question.	
Did this occur in past 6 months?	Yes / No
If yes, Impact:	None / Some / Significant
Did this occur more than 6 months ago?	Yes / No

If yes, Impact:	None / Some / Significant
12. Something you valued was lost or stolen. Yes/No. If no, go to next question.	
Did this occur in past 6 months?	Yes / No
If yes, Impact:	None / Some / Significant
Did this occur more than 6 months ago?	Yes / No
If yes, Impact:	None / Some / Significant

SECTION 9: PHYSICAL ACTIVITY – MODIFIED STEPS/GPAQ

	The next questions are about the time you spend doing different types of physical activities. This includes activities you do **at home, at work, travelling from place to place and during your spare time**. You are requested to answer the questions even if you don't consider yourself to be an active person.		
	**Occupation-Related Physical Activity (paid or unpaid work):** When answering the following questions, think back over the **past 12 months** and consider (think of) **a usual week**.		
9A	Does your work involve vigorous activities, (like heavy lifting, digging, or heavy construction)		
	for **at least 9 minutes** at a time?	Yes<br>1	No<br>0
	**If No, go to question 9D**		
9B	In **a usual week**, how many days do you do vigorous activities as part of your work?		

Participant Number

	DAYS:				
9C	On **a usual day** on which you do vigorous activities, how much time do you spend doing such work?				
	1	HOURS:			
	2	MINUTES:			
9D	Does your work involve moderate-intensity activities, (like brisk walking or carrying light loads)				
	for **at least 9 minutes** at a time?			Yes<br>1	No<br>0
	**If No, go to question 9G**				
9E	In **a usual week**, how many days do you do moderate-intensity activities as part of your work?				
	Days:				
9F	On **a usual day** on which you do moderate-intensity activities, how much time do you spend doing such work?				
	1	Hours:			
	2	Minutes:			
	**Travel-related physical activity:** other than activities that you've already mentioned, i would like to ask you about the way you travel to and from places (to work, to shopping, to market, to church, etc.).				
9G	Do you walk or use a bicycle (pedal cycle) for **at least 9 minutes** at a time to get to and from places?				
				Yes<br>1	No<br>0
	**If no, go to question 9J**				
9H	In **a usual week**, how many days do you walk or cycle for at least 9 minutes to get to and from places?				
	Days:				
9I	On **a usual day,** how much time do you spend walking or cycling for travel?				

	1	Hours:			
	2	Minutes:			
	**Non-work related and leisure time physical activity:** the next questions ask about activities you do in your leisure or spare time, for recreation or fitness**.** Do not include the physical activities you do at work or for travel already mentioned.				
9J	In your leisure or spare time, do you do any vigorous activities (like running or strenuous				
	Sports, weightlifting) for **at least 9 minutes** at a time?			Yes<br>1	No<br>0
	**If no, go to question 9M**				
9K	In **a usual week**, how many days do you do vigorous activities as part of your leisure or spare time?				
	Days:				
9L	How much time do you spend doing this on **a usual day?**				
	1	Hours:			
	2	Minutes:			
9M	In your leisure or spare time, do you do any moderate-intensity activities (like brisk walking, cycling or				
	Swimming) for **at least 9 minutes** at a time?			Yes<br>1	No<br>0
	**If no, go to question 9P**				
9N	In **a usual week**, how many days do you do moderate-intensity activities as part of your leisure				
	Or spare time?<br>Days:				
9O	How much time do you spend doing this on **a usual day?**				
		Hours:			
		Minutes:			
	**Sitting / resting activity:** now I would like to ask you about the time spent sitting or resting, not including sleeping, **in the past 7 days**. This may include time sitting at a desk, visiting friends, reading, or sitting down to watch television **during working hours and leisure or spare time**.				
9P	Over the **past 7 days,** how much time did you spend sitting or reclining (lying) on **a usual day (excluding sleeping)?**				

		Hours:			
		Minutes:			
9Q	On average, how much time, each day, do you spend watching television?				
		Hours:			
		Minutes:			

THANK YOU FOR PARTICIPATING IN THE PRO2D STUDY. HAVE A GOOD DAY
