# Supplementary material for: Progression to type 2 diabetes mellitus and associated risk factors after hyperglycemia first detected in pregnancy: A cross-sectional study in Cape Town, South Africa
Source: PLoS Med. 2019 Sep 9;16(9):e1002865. doi: 10.1371/journal.pmed.1002865 (PMC6733438; doi:10.1371/journal.pmed.1002865)
Supplement: S1 Table — (DOCX) [file pmed.1002865.s004.docx]

**S4 Table 1. Comparison of participants followed up 5-6 years later and the participants lost to follow-up**

|  |  | Not followed-up, N = 278 | Followed-up, N = 220 |
| --- | --- | --- | --- |
| Age at booking | Median (IQR) years | 31 (26-35) | 31 (27-35) |
| Number of pregnancies | Median (IQR) | 3 (2-4) | 3 (2-4) |
| Number of live births | Median (IQR) | 1 (1-2) | 1 (0-2) |
| Gestational age at booking | Median (IQR) weeks | 17 (13-22) | 15 (11-21) |
| Syphilis (VDRL positive) | n (%) | 5 (1.8) | 4(1.8) |
| HIV positive | n (%) | 19 (6.9) | 12 (5.5) |
| CD4 count | Median (IQR) cells/l | 454 (406-545) | 492.5 (364-774) |
| BMI at booking | Median (IQR) kg/m^2^ | 32.7 (27.6-38.4) | 34.6 (28.8-41.4) |
| Random blood glucose | Median (IQR) mmol/l | 12.7 (11.4-14.4) | 12.7 (11.2-14.1) |
| Fasting plasma glucose at HFDP diagnosis | Median (IQR) mmol/l | 5.9 (5.3-6.9) | 5.8 (5.1-6.5) |
| 1-hour OGTT plasma glucose at GDM diagnosis | Median (IQR) mmol/l | 10.6 (9.4-12.2) | 10.5 (9.2-11.5) |
| 2-hour OGTT plasma glucose at HFDP | Median (IQR) mmol/l | 12 (11.2-12.8) | 9 (8.2-10) |
| Treated with metformin during HFDP | n (%) | 99 (35.6) | 61 (28.4) |
| Treated with glibenclamide during HFDP | n (%) | 9 (3.3) | 7 (3.3) |
| Treated with insulin during HFDP | n (%) | 80 (28.8) | 52 (24.1) |
| Treated with both orals and insulin during HFDP | n (%) | 4 (1.4) | 3 (1.4) |
| Number of hospital admissions during HFDP | Median (IQR) | 2 (1-3) | 2 (1-3) |
| HbA_1c_ at booking | Median (IQR) % | 6.4 (5.9-7.2) | 6.5 (6-7.4) |
| Type of birth delivery | Caesarian section, n (%) | 144 (52.8) | 120 (56.3) |
|  | Normal virginal, n (%) | 129 (47.3) | 93 (43.7) |
| Fetal outcome | Stillborn or miscarriage, n (%) | 10 (3.6) | 6 (2.8) |
| Admitted to NICU | n (%) | 2 (0.7) | 3 (1.4) |
| Fetal birthweight | Median (IQR) grams | 3230 (2805-3620) | 3292 (2925-3610) |

**NICE 2008 guidelines were used. GDM referred to women with the following glucose concentrations at diagnosis: fasting>7.0mmol/l and OGTT 2-hours ≥11.1mmol/l. IGT referred to women with the following glucose at diagnosis: fasting between 5.6-6.9mmol/l and OGTT between 7.8-11.0mmol/l. in the WHO 2013 GDM diagnosis guidelines, the “GDM” group in the NICE guidelines is now the diabetes in pregnancy (DIP) group while the IGT group is now the GDM*
