# Supplementary material for: Progression to type 2 diabetes mellitus and associated risk factors after hyperglycemia first detected in pregnancy: A cross-sectional study in Cape Town, South Africa
Source: PLoS Med. 2019 Sep 9;16(9):e1002865. doi: 10.1371/journal.pmed.1002865 (PMC6733438; doi:10.1371/journal.pmed.1002865)
Supplement: S2 Table — HFDP, hyperglycemia first detected in pregnancy; T2DM, type 2 diabetes mellitus. (DOCX) [file pmed.1002865.s005.docx]

**S10 Table 2. Progression to T2DM after HFDP – studies published from 2000-2019**

| **Study** | **Sample Size** | **Response Rate** | **Country** | **Design** | **GDM Diagnosis** | **Follow Up Period** | **Progression to T2D** | **Progression to impaired glucose regulation*** | **Risk factors for progression** |
| --- | --- | --- | --- | --- | --- | --- | --- | --- | --- |
| (Lowe et al., 2018) | 4697 (GDM663) | 69.1% | Multicenter (10 countries) | Cohort | IADPSG | 11.4 years | 10.7%, | 41.5% | GDM |
| (Huvinen et al., 2018) | 333 | 95.7% | Finland | prospective cohort | WHO 1998 (5.3, 10,8.6) | 5 years | 3.6% | 15% | GDM |
| (Inoue et al., 2018) | 77 | 22.3% | Japan | retrospective cohort | IADPSG | 2 years | 22% | 44.1% | 2-hour glucose (OR 1.04), HbA1C% (OR 5.4), perinatal complication (OR 7.4), family history DM (OR 3.7) |
| (Simmons et al., 2017) | 2786 (50 previous GDM) | NR* | Maori New Zealand | cross sectional | self-reported past GDM | lifetime GDM history | 20% | NR | GDM |
| (Chamberlain et al., 2016) | 578 | NR | Australia indigenous | retrospective cohort | Australian DA, FPG ≥5.5, 2-hour ≥8 | 3, 5, 7 years | 21.9% 3 yrs, 25.5% 5 yrs, 42.4% 7 yrs | NR | early pregnancy BMI≥25(HR 3.16), partial breastfeeding at discharge (HR 2.34), 3. aboriginal status (HR4.6) |
|  | 332 |  | Australia non-indigenous |  |  |  | 4.2% 3 yrs, 5.7% 5 yrs, 13.5% 7 yrs |  |  |
| (Gupta et al., 2017) | 366 | 37% | India | Cohort | Carpenter and Coustan, IASDPSG | 14 months | 40% | 32% | high pre-pregnancy-BMI (OR1.16), acanthosis nigrins (OR3.1), post- partum screening interval (by 1 month) (OR1.02), age (OR1.1) |
| (Tam et al., 2012) | 139 (45 GDM) | 68.4 | China | prospective cohort | WHO 1998 | 15 years | 24.4% | 9.8% | Only GDM |
| (Wang et al., 2012) | 19998 (GDM =1142) | NR | USA | cohort | WHO 1998 ADA 2003 | 8.6 years | 28.7% | NR | Black American race, age, BMI |
| (Madarász et al., 2009) | 68 GDM 39NGT | 52.50% | Hungary | Follow up cohort | WHO 1985 | 4 years | 21% | 15% | FPG (OR3.4) |
| (Lee et al., 2008) | 868 GDM 868 controls | NR | Korea | case-control | National Diabetes Data Group | 2 years | 11.5% | NR | GDM (OR 3.7), family history (OR2.2), wc>85cm (OR2.3) |
| (Rivero et al., 2008) | 109 | 85.8% | Brazil | prospective cohort | ADA 1997 Working Force on DM and Pregnancy1985 | 32 months | 17.4% | 39.4% | pre-pregnancy BMI and follow-up BMI - univariate analysis |
| (Tam et al., 2007) | 203 (63 GDM) | 92.6% | China | prospective cohort | WHO 1998 | 8 years | 9% | 30.2% | Pre-pregnancy BMI≥23 (OR 3.4) |
| (Krishnaveni et al., 2007) | 555, gdm = 35 | 88% | India | cohort | Carpenter and Coustan | 5 years | 37% | 31% | GDM, WC (OR 1.1), family history (OR 10.6) |
| (Kale et al., 2004) | 126 | 69.20% | India | Follow up cohort | WHO 1985 | 4.5 years | 52% | 19% | age≥30yrsGDM (OR3.7), family history (OR2.4), 2-hour glucose>10mmol/l(OR3.6), WHR>0.85(OR2.48) |

NR* - not reported

References

CHAMBERLAIN, C. R., OLDENBURG, B., WILSON, A. N., EADES, S. J., O'DEA, K., OATS, J. J. & WOLFE, R. 2016. Type 2 diabetes after gestational diabetes: greater than fourfold risk among Indigenous compared with non-Indigenous Australian women. *Diabetes Metab Res Rev,* 32**,** 217-27.

GUPTA, Y., KAPOOR, D., DESAI, A., PRAVEEN, D., JOSHI, R., ROZATI, R., BHATLA, N., PRABHAKARAN, D., REDDY, P., PATEL, A. & TANDON, N. 2017. Conversion of gestational diabetes mellitus to future Type 2 diabetes mellitus and the predictive value of HbA1c in an Indian cohort. *Diabet Med,* 34**,** 37-43.

HUVINEN, E., ERIKSSON, J. G., KOIVUSALO, S. B., GROTENFELT, N., TIITINEN, A., STACH-LEMPINEN, B. & RONO, K. 2018. Heterogeneity of gestational diabetes (GDM) and long-term risk of diabetes and metabolic syndrome: findings from the RADIEL study follow-up. *Acta Diabetol,* 55**,** 493-501.

INOUE, H., ISHIKAWA, K., TAKEDA, K., KOBAYASHI, A., KURITA, K., KUMAGAI, J., YOKOH, H. & YOKOTE, K. 2018. Postpartum risk of diabetes and predictive factors for glucose intolerance in East Asian women with gestational diabetes. *Diabetes research and clinical practice,* 140**,** 1-8.

KALE, S., YAJNIK, C., KULKARNI, S., MEENAKUMARI, K., JOGLEKAR, A., KHORSAND, N., LADKAT, R., RAMDAS, L. & LUBREE, H. 2004. High risk of diabetes and metabolic syndrome in Indian women with gestational diabetes mellitus. *Diabetic medicine,* 21**,** 1257-1258.

KRISHNAVENI, G. V., HILL, J. C., VEENA, S. R., GEETHA, S., JAYAKUMAR, M. N., KARAT, C. L. S. & FALL, C. H. D. 2007. Gestational diabetes and the incidence of diabetes in the 5 years following the index pregnancy in South Indian women. *Diabetes research and clinical practice,* 78**,** 398-404.

LEE, H., JANG, H. C., PARK, H. K., METZGER, B. E. & CHO, N. H. 2008. Prevalence of type 2 diabetes among women with a previous history of gestational diabetes mellitus. *Diabetes research and clinical practice,* 81**,** 124-129.

LOWE, W. L., SCHOLTENS, D. M., LOWE, L. P., KUANG, A., NODZENSKI, M., TALBOT, O., CATALANO, P. M., LINDER, B., BRICKMAN, W. J. & CLAYTON, P. 2018. Association of Gestational Diabetes With Maternal Disorders of Glucose Metabolism and Childhood Adiposity. *JAMA,* 320**,** 1005-1016.

MADARÁSZ, E., TAMÁS, G., TABÁK, Á. G. & KERÉNYI, Z. 2009. Carbohydrate metabolism and cardiovascular risk factors 4 years after a pregnancy complicated by gestational diabetes. *Diabetes research and clinical practice,* 85**,** 197-202.

RIVERO, K., PORTAL, V. L., VIEIRA, M. & BEHLE, I. 2008. Prevalence of the impaired glucose metabolism and its association with risk factors for coronary artery disease in women with gestational diabetes. *Diabetes Res Clin Pract,* 79**,** 433-7.

SIMMONS, D., KUMAR, S., CROOK, N. & RUSH, E. 2017. Diabetes among Maori women with self-reported past gestational diabetes mellitus in a New Zealand Maori community. *Aust N Z J Obstet Gynaecol,* 57**,** 599-603.

TAM, W. H., MA, R. C., YANG, X., KO, G. T., LAO, T. T., CHAN, M. H., LAM, C. W., COCKRAM, C. S. & CHAN, J. C. 2012. Cardiometabolic risk in Chinese women with prior gestational diabetes: a 15-year follow-up study. *Gynecol Obstet Invest,* 73**,** 168-76.

TAM, W. H., YANG, X. L., CHAN, J. C. N., KO, G. T. C., TONG, P. C. Y., MA, R. C. W., COCKRAM, C. S., SAHOTA, D. & ROGERS, M. S. 2007. Progression to impaired glucose regulation, diabetes and metabolic syndrome in Chinese women with a past history of gestational diabetes. *Diabetes/metabolism research and reviews,* 23**,** 485-489.

WANG, Y., CHEN, L., HORSWELL, R., XIAO, K., BESSE, J., JOHNSON, J., RYAN, D. H. & HU, G. 2012. Racial differences in the association between gestational diabetes mellitus and risk of type 2 diabetes. *J Womens Health (Larchmt),* 21**,** 628-33.
